# The Evaporative Function of Cockroach Hygroreceptors

**DOI:** 10.1371/journal.pone.0053998

**Published:** 2013-01-16

**Authors:** Harald Tichy, Wolfgang Kallina

**Affiliations:** Department of Neurobiology, Faculty of Life Sciences, University of Vienna, Vienna, Austria; University of Arizona, United States of America

## Abstract

Insect hygroreceptors associate as antagonistic pairs of a moist cell and a dry cell together with a cold cell in small cuticular sensilla on the antennae. The mechanisms by which the atmospheric humidity stimulates the hygroreceptive cells remain elusive. Three models for humidity transduction have been proposed in which hygroreceptors operate either as mechanical hygrometers, evaporation detectors or psychrometers. Mechanical hygrometers are assumed to respond to the relative humidity, evaporation detectors to the saturation deficit and psychrometers to the temperature depression (the difference between wet-bulb and dry-bulb temperatures). The models refer to different ways of expressing humidity. This also means, however, that at different temperatures these different types of hygroreceptors indicate very different humidity conditions. The present study tested the adequacy of the three models on the cockroach’s moist and dry cells by determining whether the specific predictions about the temperature-dependence of the humidity responses are indeed observed. While in previous studies stimulation consisted of rapid step-like humidity changes, here we changed humidity slowly and continuously up and down in a sinusoidal fashion. The low rates of change made it possible to measure instantaneous humidity values based on UV-absorption and to assign these values to the hygroreceptive sensillum. The moist cell fitted neither the mechanical hygrometer nor the evaporation detector model: the temperature dependence of its humidity responses could not be attributed to relative humidity or to saturation deficit, respectively. The psychrometer model, however, was verified by the close relationships of the moist cell’s response with the wet-bulb temperature and the dry cell’s response with the dry-bulb temperature. Thus, the hygroreceptors respond to evaporation and the resulting cooling due to the wetness or dryness of the air. The drier the ambient air (absolutely) and the higher the temperature, the greater the evaporative temperature depression and the power to desiccate.

## Introduction

Humidity influences the survival of insects mainly by affecting their water content. If humidity can be kept within certain limits, exposure to dry or humid conditions may not be harmful. Insects are capable of maintaining a stable water balance by searching for a suitable environment. Humidity choice responses depend on the existence of hygroreceptive sensilla, as has been demonstrated by experimentation in several species. Externally, these sensilla appear as small cuticular pegs originating from the antennal surface or set in pits. They house two types of hygroreceptive cells which respond antagonistically to changes in humidity. The rate of discharge of one type is increased by moist air and decreased by dry air. These cells have been labeled “moist” cells, a terminology maintained in the present paper. The discharge rate of the second type, labeled “dry” cells, is increased by dry air and decreased by moist air. Both types of hygroreceptive cells occur together in the same sensillum along with a thermoreceptive “cold” cell type [1], [2], [3], [4], [5], [6].

**Figure 1:**
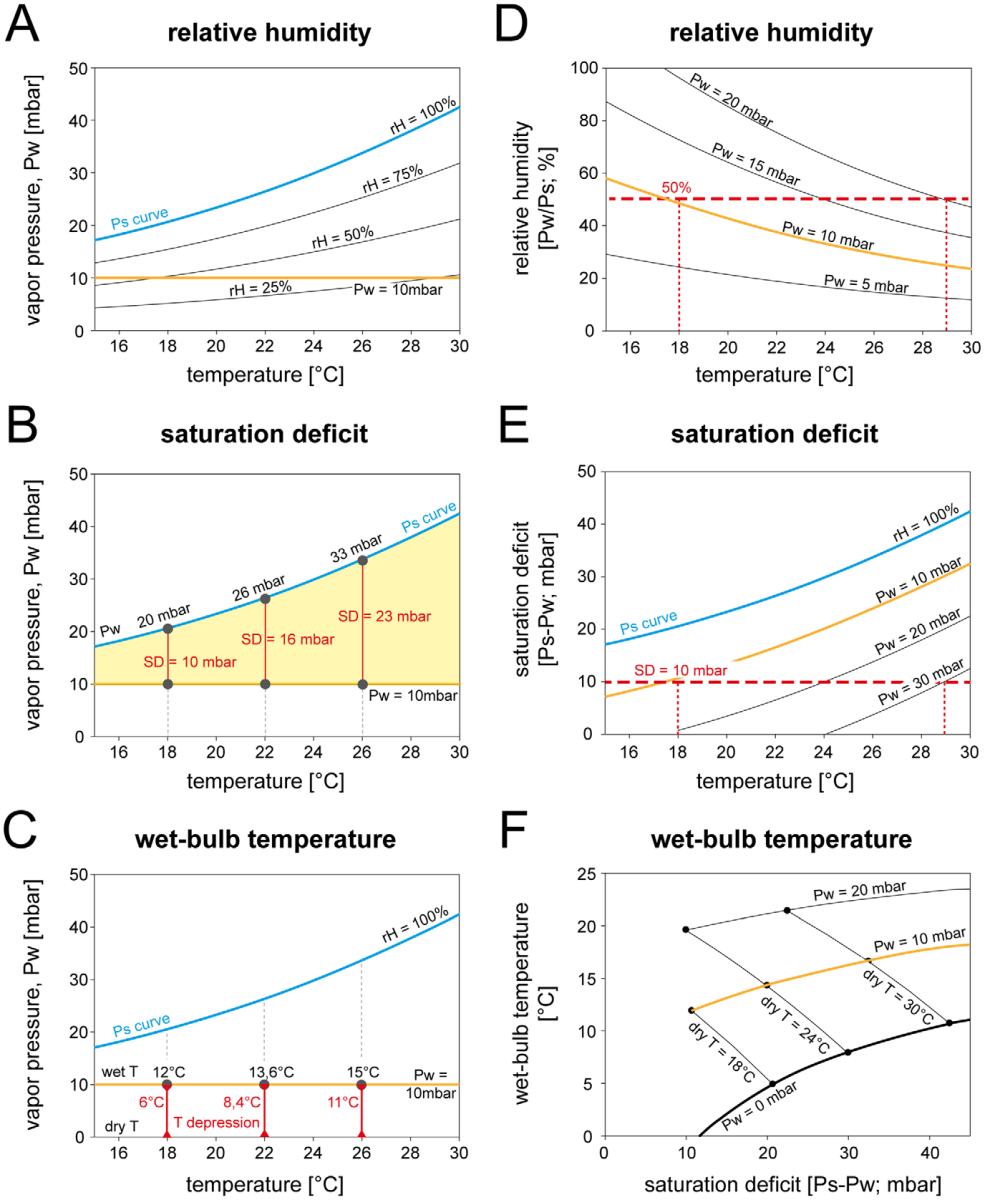
Calculated relationships between the key parameters of humidity and atmospheric temperature. **A**. Relationship between vapor pressure, relative humidity and temperature. The relative humidity is the ratio of vapor pressure and saturation vapor pressure times 100. As the saturation vapor pressure increases with rising temperature, the relative humidity decreases when the vapor pressure is constant. **B**. Relationship between vapor pressure, saturation deficit and temperature. The saturation deficit is the difference between vapor pressure and saturation vapor pressure and shows the amount of water vapor required for saturation at different temperatures. As the saturation vapor pressure increases with rising temperature, the saturation deficit increases when the vapor pressure is constant. **C**. Relationship between vapor pressure, wet-bulb temperature and dry-bulb temperature, which is the atmospheric temperature. Temperature depression is the difference between wet-bulb and dry-bulb temperature. As the saturation vapor pressure increases with rising temperature, the wet-bulb temperature as well as the temperature depression increases when the vapor pressure is constant. **D**. Relationship between relative humidity, vapor pressure and temperature. **E**. Relationship between saturation deficit, vapor pressure and temperature. **F**. Relationship between wet-bulb temperature, vapor pressure and saturation deficit. At constant vapor pressure (*orange line*), both the wet-bulb temperature and the saturation deficit increases with rising (dry-bulb) temperature. *Pw* water vapor pressure, *Ps* saturation water vapor pressure, *rH* relative humidity, *SD* saturation deficit, *dry T* dry-bulb temperature, *wet T* wet-bulb temperature.

Attempts to elaborate unifying concepts for the mechanism of humidity transduction have yielded three main models that require further development and refinement [1], [2], [4], [7], [8], [9], [10]. In these models, hygroreceptors are proposed to operate either as 1) mechanical hygrometers in which activity is initiated by swelling and shrinking of hygroscopic sensillum structures, 2) evaporimeters in which the rate of evaporation of water due to the dryness of the air leads to quantitative changes in the lymph concentration, and 3) psychrometers in which the degree of cooling during evaporation of water is used to measure the humidity (or the dryness) of the air. These models pose some intriguing questions as to the adequate stimulus. If it is assumed that temperature does not affect the hygroreceptors per se, it follows that hygroreceptors possessing these different transduction mechanisms would respond in different ways when tested with humidity changes at different temperatures. Hygroreceptors acting as mechanical hygrometers would respond to the relative humidity of the air (i.e., the ratio of the actual vapor pressure to the saturation water vapor pressure; Fig. 1*A*) independently of the ambient temperature. Evaporation rate detectors would respond to the saturation deficit of the air (i.e., the difference between the actual vapor pressure and the saturation water vapor pressure, given by the vertical lines in Fig. 1*B*). In psychrometers, hygroreceptors are functioning as wet-bulb and dry-bulb thermometers which determine the temperature depression due to the cooling effect of water evaporating from the sensillum surface (Fig. 1*C*).

The mechanical hygrometer model, most favored for humidity transduction, was developed based on the close association of the dendritic membranes with the sensillum wall [1], [2]. Supporting evidence for a mechanical function came from the effect of moving the recording electrode (in the cockroach [8] and in the cricket [11]) or exerting pressure to the tip of the sensillum (in the noctuid moth [12]). Both forms of mechanical stimulation can influence the firing rate of the moist and dry cells. These findings contradict related attempts to modulate the hygroreceptors discharge rates in a reversible manner by slight lateral movements of the recording electrode in both the cockroach [13] and the stick insect [14]. A more quantitative approach to demonstrate humidity-dependent swelling and shrinking of the sensillum wall involved high-resolution scans of the fine surface structures of the apical region of hygroreceptive sensilla in the honey bee using atomic force microscopy [15]. However, no change in the dimensions of the sensillum wall became apparent when ambient humidity was set through a wide range of different levels.

**Figure 2:**
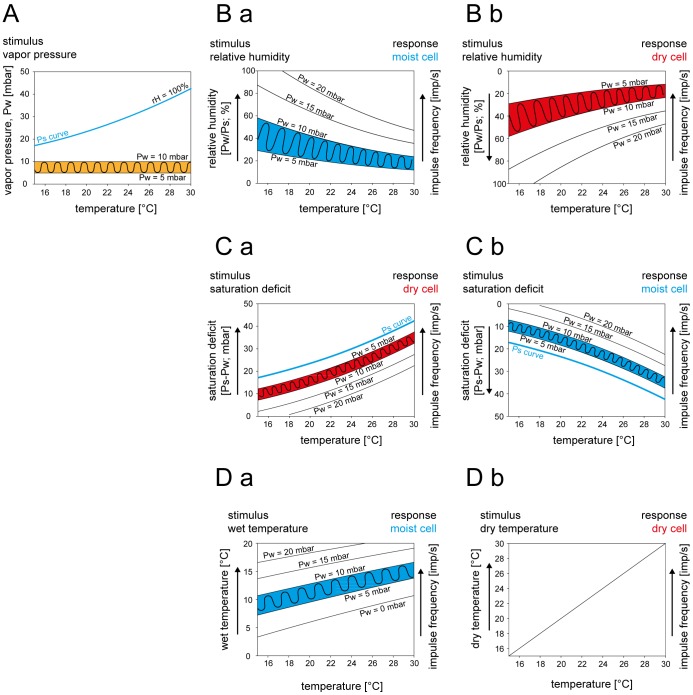
Effects of atmospheric temperature on the hygroreceptors responses as predicted by the three humidity transduction models. **A**. Humidity stimulation consists of constant amplitude oscillating change in vapor pressure, illustrated by the orange zone. **Ba**. The constant amplitude oscillating change in vapor pressure in *A* produces, with rising temperature, continuously deceasing oscillations in relative humidity (*left axis*), illustrated by the blue zone. Impulse frequency of a moist cell responding to oscillations in relative humidity is predicted to oscillate within blue zone (*right axis*). **Bb**. Same plot as in *Ba* but with turned y-axis (*left axis*) to illustrate the relative humidity stimulus eliciting excitatory responses in a dry cell. Impulse frequency of a dry cell responding to oscillations in relative humidity is proposed to oscillate within red zone (*right axis*). **Ca**. Constant amplitude oscillating change in vapor pressure in *A* produces, with rising temperature, continuously increasing oscillations in saturation deficit (*left axis*), illustrated by the red zone. Impulse frequency of a dry cell responding to oscillations in saturation deficit is predicted to oscillate within red zone (*right axis*). **Cb**. Same plot as in *Ca* but with turned y-axis (*left axis*) to illustrate the saturation deficit stimulus eliciting excitatory responses in a moist cell. Impulse frequency of a moist cell responding to oscillations in saturation deficit is predicted to oscillate within blue zone (*right axis*). **Da**. Constant amplitude oscillating change in vapor pressure in *A* produces, with rising temperature, continuously increasing oscillations in wet-bulb temperature (*left axis*), illustrated by the blue zone. Impulse frequency of a moist cell responding to oscillations in wet-bulb temperature is predicted to oscillate within blue zone (*right axis*). **Db**. Dry-bulb temperature as function of air temperature. Impulse frequency of a dry cell responding to the dry-bulb temperature is predicted to increase with rising temperature (*right axis*). *Pw* water vapor pressure, *Ps* saturation water vapor pressure. *Arrows* point in the direction of increasing axis values.

The present study was designed to assess the adequate stimulus of the cockroach’s moist and dry cells by determining whether the responses predicted from the three models of humidity transduction are indeed observed. A basic problem of earlier investigations of humidity transduction was the lack of precise information on the stimulus input. Standard hygrometers were too slow to measure humidity during the rapid changes utilized to test the validity of the mechanical model [4], [7], [8]. It was therefore impossible to determine the rate of humidity change of the stimulating air stream and determine reasonably exact measurements from the sensillum when the humidity was changing. The problem was solved in the present study by changing the vapor pressure at low rates and by measuring instantaneous values of vapor pressure by means of an UV-absorption hygrometer at a rate of 100 Hz. An effort was made to produce constant-amplitude sinusoidal changes in vapor pressure. One advantage of this system was the repeated measurement at different temperatures (Fig. 2*A*, shaded area). Expressing the constant-amplitude vapor pressure oscillations as oscillations in the relative humidity, in the saturation deficit or in the wet-bulb temperature should make it possible to determine which of these humidity oscillations adequately explain the responses of the moist and dry cells. With rising temperature, the oscillations in the relative humidity slightly decrease in amplitude and mean value (Fig. 2*B* *a*), while the saturation deficit oscillates with constant amplitude and the mean value sharply increases (Fig. 2*C* *a*). The oscillating wet-bulb temperature, in contrast, decreases slightly in amplitude but the mean value increases moderately with rising temperature (Fig. 2*D* *a*).

What is expected from a hygroreceptor that operates as mechanical hygrometer, evaporation detector or psychrometer? The mechanical hygrometer model assumes that moist and dry cells respond to the relative humidity of the atmosphere independently of its temperature. Therefore, the oscillating discharge rates should follow the oscillations in relative humidity. Since the humidity coefficient is positive in moist cells and negative in the dry cells, the moist cell’s oscillating impulse frequency should decrease with rising temperature (shaded area in Fig. 2*B* *a*; impulse frequency is drawn on right axis of the diagram) and the dry cell’s oscillating impulse frequency should increase with rising temperature (shaded area in Fig. 2*B* *b*). The evaporation model, in contrast, assumes that moist and dry cells respond to the saturation deficit of the atmosphere. Therefore, the moist cell’s oscillating discharge rates should decrease with rising temperature (Fig. 2*C* *b*). Due to the dry cell’s negative humidity coefficient, its oscillating impulse frequency should increase with rising temperature (Fig. 2*C* *a*). The psychrometer model proposes that the moist cell is a wet-bulb thermometer that measures the degree of cooling due to evaporation and the dry cell is the dry-bulb-thermometer that measures the actual temperature. Accordingly, the moist cell’s discharge rates should oscillate and thereby increase with rising temperature (Fig. 2*D* *a*) and the dry cell’s discharge rates should not oscillate but also increase with rising temperature (Fig. 2*D* *b*).

The results of our analysis show variance with both the mechanical and the evaporation model but support the psychrometer model in which evaporative cooling due to the vapor pressure gradient between the sensillum surface and the atmosphere is the adequate stimulus.

## Methods

### Animals and Preparation

The adult male cockroaches, *Periplaneta americana*, used in this study were obtained from a crowded colony which was maintained on a 12∶12 dark/light cycle at temperatures between 20°C and 26°C. Only animals with antennae exceeding 50 mm in length were used. Thus, the flagellum extended 20 mm beyond the segments from which the recordings from hygroreceptive sensilla were made. The cockroach was fixed to a holder with adhesive tape, and one antenna was attached to a narrow support with dental cement (Harvard Cement) for unobstructed humidity stimulation. The hygro-thermoreceptive sensilla are located on the middle antennal region only on its ventral side near the distal margin of the ring-shaped segments. When viewed from an angle of roughly 60° to the antenna, the sensillum showed up as bright point among small hair-shaped sensilla on a brown surface. Final identification came from the responses to humidity stimulation. Action potentials were recorded between two electrolytically sharpened tungsten wires, one inserted at an angle of about 45° into the sensillum base, and the other lengthwise about 2 mm into the tip of the antenna. After amplification, the band pass (0.1–3 kHz) filtered signals were passed through an AD-converter (1401 plus, Cambridge Electronic Design; 12-bit; 10 kHz) and fed into a PC for online recording. The digitized impulses and the voltage output of the electronic flow meters were displayed on-line on a monitor, stored on a hard disk and sorted off-line using Spike2 software (Cambridge Electronic Design, UK). Spike parameters were extracted from the stored waveform channel and sampled to form templates. Detected spikes were then subjected to the template-matching system to create or modify the templates. Each spike was compared against the templates and each time a template was confirmed it was added to the template by overdrawing. Adding a spike to a template may change the shape and width of the template outlines. Thus the template boundaries display homogeneity of classification.

### Response Evaluation

Impulse frequency (*F*, impulses/s) was calculated from running averages of three consecutive 0.5-s intervals. In case of dry cells’ responses to the dry-bulb temperature, the variation of impulse frequency in a single oscillation period was found by calculating the mean value and the standard deviations of all 0.5-s intervals of that period. Probably the most important characteristics of a sensory cell are the gain and the resolving power.

### Gain of Response

The gain of the hygroreceptor’s response refers to the change in impulse frequency per unit change in humidity. This quantity is given by the slope of the function that approximates the relation between stimulus and excitation. Because of the double dependence of the impulse frequency on the instantaneous humidity and the rate with which the humidity changes, the approximation was done by least-squares multiple regressions which took the following form: *F* = *y_0_*+*a dx/dt*+*b* x, in which *F* is the impulse frequency, *y_0_* is the height of the regression plane or its intercept with the *F* axis, *a* is the gain for the rate of humidity change expressed as relative humidity (impulses/s)/(%rH/s), saturation deficit (impulses/s)/(mbar/s) or wet-bulb temperature (impulse/s)/(°C/s), and *b* is the gain for the instantaneous humidity expressed as relative humidity (impulses/s)/%rH, saturation deficit (impulses/s)/mbar or wet-bulb temperature (impulses/s)/°C. The *y_0_* intercept is the estimated average value of the impulse frequency when the instantaneous humidity and its rate of change are equal to zero (or the value of *y* when *x* is 0). It defines the elevation of the regression plane and thus the dependence of the hygroreceptor’s response magnitude on the temperature level at which the humidity stimulation takes place. The negative *y_0_* intercept values of the moist cell’s responses to wet-bulb stimuli do not indicate negative discharges. Since *x* was never 0°C, the *y_0_* intercept at 0°C is no intrinsic parameter of the regression plane. When moving the position of the *y* axis along the *x* axis to the actual temperature range between 16 and 28°C, the *y_0_* intercept values become positive and indicate the mean *y* values at the value chosen for *x*. Nonetheless, the negative *y_0_* intercept values were applied to the regression analysis because the chief concern here was not to compare the response magnitudes at a specific temperature but to compare the relationship between the response magnitude and the temperature. For this question, the sign of the *y_0_* intercept values is not relevant.

The *R* ^2^ coefficient of determination, indicating how well the regression plane approximates the real data points, was interpreted as the proportion of response variation explained by the regression plane. Statistical analyses and plots were performed with the SigmaPlot 10.0 software (Systat, Inc., San Jose, California, USA).

### Resolving Power

From the gain of responses and the scatter of individual responses, the resolving power of a hygroreceptor cell was determined by the maximum number of discrete steps that the impulse frequency can distinguish within a temperature range. To estimate the step numbers of a hygroreceptor cell, above and below the frequency-vs-stimulus-curve another curve was plotted which encloses the deviation of the responses throughout the range. Such a band reflects the degree of scatter. The stimulus steps were then drawn within the space enclosed by the deviations. Step width (Δ*x*) reflects resolving power.

In addition to such a graphical approach, resolving power was derived directly from the experimental data. Attention focused on a hygroreceptor cell at average gain and a single pair of responses, one to each of two temperatures. The question posed was: “For the larger response to be associated with the higher temperature with a specific high degree of probability, e.g. 90%, how different must the temperatures be?” A full mathematical development of the concepts underlying the resolving power (Δ*x*) was presented by Loftus and Corbière-Tichané [16]. The equation is
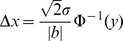
in which |*b*| is the mean absolute slope of the stimulus-response functions, σ^2^ is the variance of the individual deviations of points about their respective regressions, *γ* is the required probability (90%), and Φ^−1^ *_(γ)_* is the inverse of the distribution function of a standardized, normally distributed, random variable, Φ^−1^ *_(0.9)_*_ = _1.28 ([17], Tables p. 28). σ^2^ is estimated by



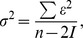
where *ε* is the deviation of each individual point from its curve, *I* is the number of curves, and *n* the number of measurements. *n* is reduced by the number of degrees of freedom, which is *2I* because 2 estimates are necessary to determine each straight line (*a* and *b; y = a+bx*).

This method can be applied if the following conditions are met: (*i*) the deviations of the individual points from their curves must be normally distributed about a mean of zero, and (*ii*) the absolute deviations (sign ignored) must not depend on the slope of the curves. The absolute deviations of single points from their regressions did not depend on the slopes of the regressions. Their distribution, however, was not normal (x^2^-test*)*. Though bell-shaped, the flanks of the distribution curve were too steep; the points tended to be located too centrally. This type of distribution will, if anything, underestimate the resolving power. The normal distribution model was accepted for the lack of a better one.

### Control of Humidity and Temperature

**Figure 3:**
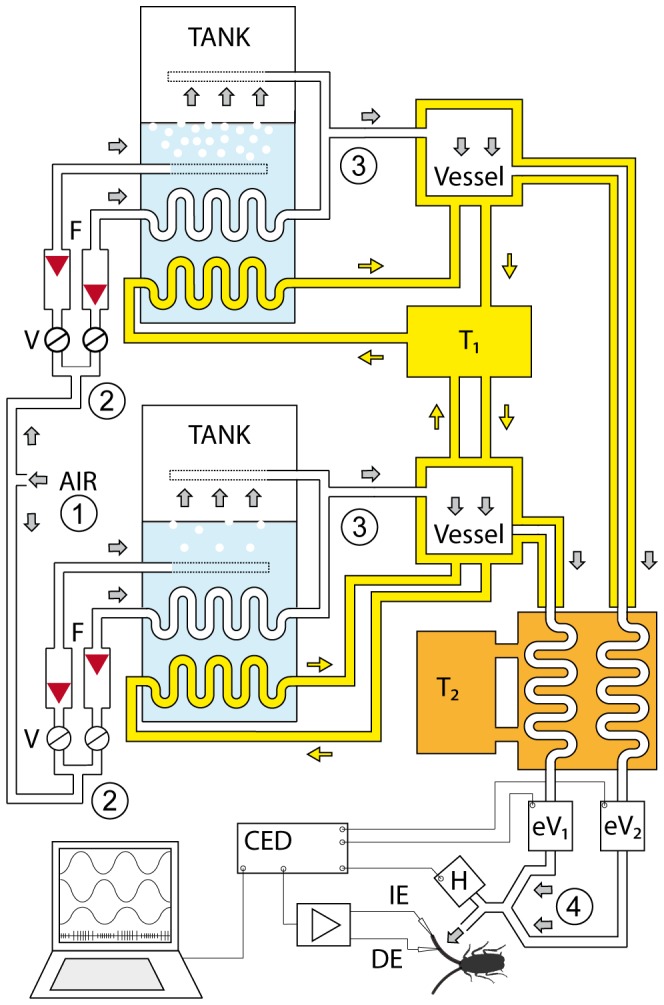
Delivery system for sinusoidal humidity modulation. Compressed air was divided at *1* into two air streams to be set at different water vapor pressure values. Each stream was further split into two substreams (at *2*) and their flow rates adjusted by needle valves (*V*) and monitored continuously by flow meters (*F*) before passing through a *tank* of water at constant depth and temperature (42°C). One substream bubbled out through many holes in a polyethylene tubing in a tank of ion-exchange water at constant 42°C. Temperature was controlled by thermostat 1 (*T_1_*). The second substream was conducted through the spiral tube in the same tank but remained dry when it was also warmed to 42°C. After emerging from the tank, the two substreams were combined in a single stream (at *3*) variable in water vapor content from dry to almost saturated. Homogeneity of mixture was enhanced by a 2-l series connected *vessel*. Water-jacket insulation is shown. The temperature of the two air streams was then set at given temperature levels by driving them through a thermostatically controlled heat exchanger (*T_2_*). After passing through electrical proportional valves (eV_1_, eV_2_), the air streams were combined to a single stream (at *4*). The vapor pressure of his stream was sinusoidally modulated by mixing the two streams in a ratio determined by the proportional valves by means of the output sequencer function of the data acquisition software (Spike2; Cambridge Electronic Design, *CED*, Cambridge, UK). By 180° phase shifting of the control voltages of the electrical proportional valves, the flow rate of the combined air stream was held constant. The antenna was placed at the outlet of the stimulus air stream, the recording or different electrode (*DE*) inserted at the base of the hygroreceptive sensillum and the reference or indifferent electrode (*IE*) into the tip of the antenna. Humidity stimulation was measured by a UV-absorption hygrometer (*H*).

Air from a pressure-regulated source was cleaned, dried and split into two streams (Fig. 3). Their flow rates were equalized by matching the rates in mass flow meters. The first stream was bubbled out through many openings in a polyethylene tube firmly anchored in a tank containing ion-exchange purified water at constant depth and a temperature of 42°C. The second stream was conducted through the tank in a spiral tube and remained dry as it was warmed to 42°C. The temperature of the two streams was then set at different temperature levels by driving them through a further self-made, thermostatically controlled heat exchanger. After emerging from the heat exchanger, the two air streams passed through electrical proportional valves (KWS 3/4, Kolvenbach) and then were combined to a single stream. The water vapor pressure of this stream was sinusoidally modulated by mixing the two streams in a ratio determined by the proportional valves. To hold the flow rate of the mixed air constant at 2.5 m/s, the control voltages (AD-converter, 1401 plus, Cambridge Electronic Design) of the proportional valves were phase shifted by 180°. The mixed air was divided into two streams. For stimulation, the first stream was directed towards the antenna by way of a Plexiglas tube 7 mm in diameter. The hygroreceptive sensillum was 5 mm away from the outlet of the tube. By passing the second stream through a 1-cm^3^ detection chamber of an UV-absorption hygrometer (K 20, Campbell Scientific), water vapor density was measured at a rate of 100 Hz. The temperature within the first air stream was measured within ±0.03°C by a small thermistor (250×400 µm; Fenwall Electronics, BC 32 L1) 3 mm downstream from the sensillum. The voltage outputs of the hygrometer and the thermistor were passed through the AD-converter (1401 plus, Cambridge Electronic Design), fed into the PC and recorded online. Based on the digitized signals of the hygrometer and the thermistor, the vapor pressure (*Pw*) and the relative humidity (*rH*) were monitored offline. The saturation water vapor (*Ps*), the saturation deficit (*SD*) and the wet-bulb temperature (*Twet*) were calculated by using the Vaisala Humidity Calculator, a web-based software tool. The hygroreceptive sensillum was exposed to slow and continuous rising and falling humidity at rates between 1% and 5%rH/s. An effort was made to produce sinusoidal humidity changes. The obvious advantage was the repeated measurement of the discharge rates under nearly identical stimulus conditions.

During oscillating changes in vapor pressure, the temperature of the airstream displayed slight fluctuations simultaneously with the oscillations in vapor pressure. Temperature was termed constant when changes larger than 0.5°C failed to develop in the course of an oscillation period. At 24°C and 10 mbar, a change of 0.5°C produces a 1% change in the relative humidity, a 0.9 mbar change in the saturation deficit or a 0.2°C change in the wet-bulb temperature. These values were within the limits of adjustment of the vapor pressure, which determined the humidity parameters. Furthermore, such low rates have never been observed to affect the discharge rates of any insect moist or dry cell. Nonetheless, these temperature fluctuations tended to drift during a series of humidity oscillations. Such drifts were presumed to have little, if any, effect on the humidity responses of the moist and dry cells. But they affect the dry cell’s discharge rate when plotted against the dry-bulb temperature. Thus, the dry cell’s impulse frequency at any temperature level probably displayed a degree of scatter greater than if the temperatures were more stationary. As a consequence the value obtained for the dry cell’s resolving power for steady dry-bulb temperatures would be poorer than deserved.

### The Concept of the Adequate Stimulus of Insect Hygroreceptors

The data from previous research on hygroreceptive cells of different insects were remarkably consistent with (but do not prove) the mechanical hygrometer model which suggests that a hygroscopic sensillum wall attains moisture equilibrium with the relative humidity of the air [3], [4], [5], [6], [7], [8]. If this is so, and if the moist and the dry cell do respond to the relative humidity, then the sensory input from these hygroreceptors would not directly cause the behavioral reactions to the humidity gradient but must be modified at the level of the central nervous system. This is because the relative humidity is not a direct measure of any absolute quantity of the water vapor but merely a ratio between two known values. Identical relative humidity values do not indicate identical atmospheric moisture conditions unless the temperature is also the same. A 50% relative humidity at low temperatures contains much less water vapor than a 50% relative humidity at high temperatures (see intersections of the horizontal 50% line with the vapor pressure curves in Fig. 1*D*). The reason is that the higher the temperature, the more thermal energy is in a parcel of air and the more evaporative work can be done in that air parcel. Relative humidity expresses how much of the thermal energy available for evaporation has actually been used to evaporate water. A relative humidity of 50% means that half of the available energy has been used for evaporation and 50% is still available to do more work of evaporation if more molecules of water were available. The relative humidity is thus a useful measure of evaporation provided that moisture conditions are being assessed at a single constant temperature. If observations are to be extended over a range of temperatures, however, the saturation deficit or evaporative cooling are the exact measures.

Fig. 1*D* illustrates the variation in the vapor pressure and the saturation deficit at constant relative humidity values when the temperature is varied. A 50% relative humidity at 18°C is equivalent to a saturation deficit of 10.4 mbar (saturation vapor pressure = 20.7 mbar; water vapor pressure = 10.3 mbar; vertical dotted line at 18°C in Fig. 1*D*), while the relative humidity of 50% at 29°C is equivalent to a greater saturation deficit of 20.1 mbar (40.2–20.1; vertical dotted line at 29°C in Fig. 1*D*) and an increased evaporation. Evaporation of water from the sensillum surface doubles in an atmosphere of 50% relative humidity when the air temperature rises from 18 to 29°C (see intersections of the horizontal 50% line with the vapor pressure curves in Fig. 1*D*). Thus at constant relative humidity, evaporation is much greater at higher than at lower temperatures. To keep evaporation constant, an insect must search for areas of constant saturation deficit rather than constant relative humidity. Fig. 1*E* shows the variation in vapor pressure necessary to maintain a saturation deficit at a constant value when the temperature is varied. A saturation deficit of 10 mbar at 18°C is equivalent to a vapor pressure of 10.7 mbar, while the saturation deficit of 10 mbar at 29°C is equivalent to a three times greater vapor pressure of 30.2 mbar (see intersections of the horizontal saturation-deficit line with the vapor pressure curves in Fig. 1*E*).

The fact that two different areas have the same relative humidity does not imply a similar drying power of the atmosphere unless the temperatures are identical. In contrast, areas having the same saturation deficit do influence evaporation rates in the same way, whether the temperatures are identical or not. Relative humidity alone gives no indication of the evaporation rate, while the saturation deficit alone gives an indication of the evaporation rate. The saturation deficit therefore constitutes the exact indication of the drying power of the atmosphere at any temperature.

Evaporation is always accompanied by a cooling effect, which is for example indicated by the wet-bulb temperature of a moistened wick on the bulb of a thermometer exposed to the air flow. The lower the vapor pressure, the more water evaporates to cool the wet-bulb thermometer. Since evaporation increases at a constant vapor pressure when the temperature rises, the cooling effect also increases with rising temperature. Fig. 1*C* shows the variation in the wet-bulb temperature at a constant vapor pressure when the temperature is varied. For example, a vapor pressure of 10 mbar at 18°C produces a wet-bulb temperature of 12°C, whereas a vapor pressure of 10 mbar at 26°C causes a wet-bulb temperature of 15°C. The close relation between the wet-bulb temperature and the saturation deficit is illustrated in Fig. 1*F*. As indicated by dry-temperature lines, the higher the wet-bulb temperature is at constant temperature the lower the saturation deficit (i.e., the smaller the saturation deficit, the smaller the cooling effect due to evaporation). The vapor-pressure lines, in contrast, show that the wet-bulb temperatures are higher the higher the saturation deficit. This is because the dry-temperature is raised, providing more energy for reaching adiabatic saturation at the wet-bulb thermometer. At a given dry-temperature, however, each wet-bulb temperature value corresponds with a single saturation deficit value. Therefore, the dry-temperature must be known to determine the humidity (or the dryness) of the air based on the wet-bulb temperature. Together, the degree of evaporation cooling and the atmospheric temperature are a precise measure of the atmospheric humidity.

## Results

**Figure 4:**
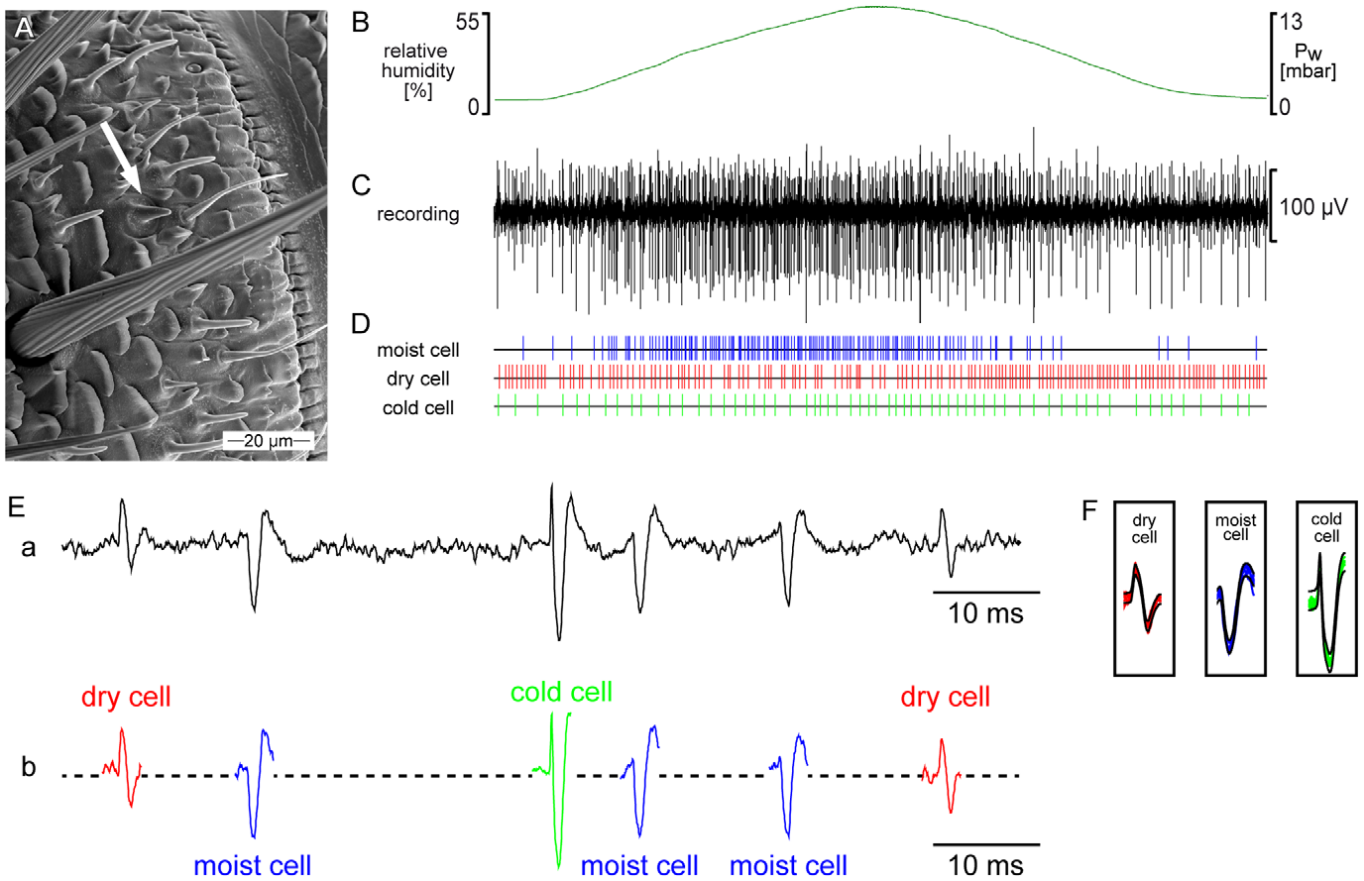
Simultaneously recorded activity of a moist cell, a dry cell and a cold cell. **A**. Scanning electron micrograph of the distal margin of a ring-shaped segment from the middle antennal region of the male cockroach showing location and external features of the hygro-thermoreceptive sensillum (*arrow*). **B**. Time course of humidity; oscillation in the relative humidity produced by oscillating changes in vapor pressure at 20.2°C. **C**. Action potentials recorded by inserting an electrode into the sensillum base. **D**. Responses of the moist cell, the dry cell and the cold cell classified off-line by the spike detecting and template matching systems of the Spike2 software (Cambridge Electronic Design, UK) and represented as raster plots. **Ea**. Detail of the recording in *C* showing action potentials of the 3 cells; **b**. Classified action potentials, obtained by matching the shape of each action potential against shape templates. The medium-sized impulses are produced by the moist cell, the small impulse amplitudes by the dry cell and the large impulses by the cold cell. **F**. Template windows showing the template boundaries of the spike waveforms from the 3 cells. *Pw* water vapor pressure, *rH* relative humidity.

A moist cell and a dry cell are combined with a cold cell in the same peg-shaped sensillum on the antenna of the cockroach (Fig. 4*A*). This triad of receptor cells typically discharged continuously when the humidity as well as the temperature of the stimulating air stream were constant. Rising humidity increased the impulse frequency of the moist cell and decreased that of the dry cell, and conversely, falling humidity increased the impulse frequency of the dry cell and decreased that of the moist cell (Fig. 4*B–D*). Under the same condition, the cold cell showed a very slight change in impulse frequency, difficult to confirm as a significant response.

### Double Dependence

**Figure 5:**
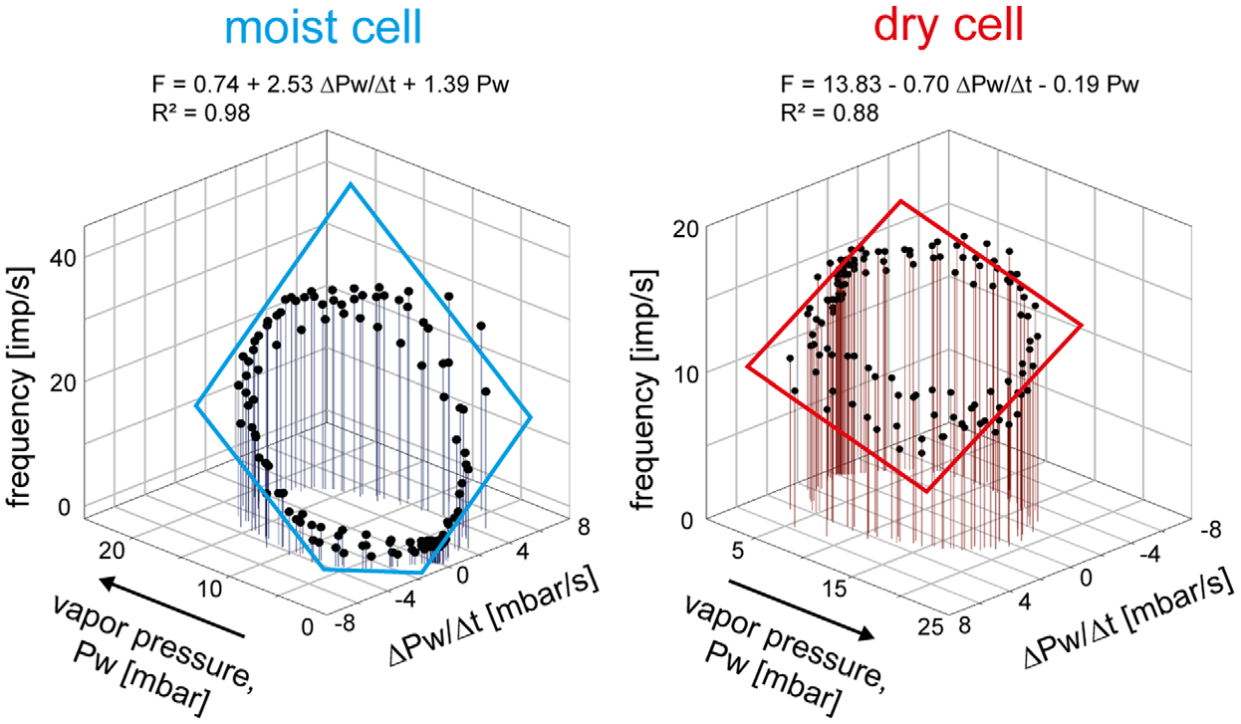
Humidity stimulation expressed as vapour pressure. Impulse frequency of a moist cell and a dry cell located in the same sensillum during three consecutive oscillations in vapor pressure as a function of instantaneous vapor pressure and its rate of change. Multiple regressions which utilize three-dimensional planes [*F* = *yo*+*a* (Δ*Pw*/Δt)+*bPw*; where *F* is the impulse frequency and *yo* is the intercept of the regression plane with the *F* axis reflecting the height of the regression plane] were calculated to determine the gain of the responses for the instantaneous vapor pressure (*b*-slope) and its rate of change (*a*-slope). Impulse frequency of the moist cell increases linearly with rising instantaneous vapor pressure and its rate of change, in the dry cell with falling instantaneous vapor pressure and its rate of change. *R^2^*, coefficient of determination; the number of points per plot is 130. *Arrows* point in the direction of increasing axis values. *F* impulse frequency, *Pw* water vapor pressure.

During slowly oscillating changes in vapor pressure the moist cell and the dry cell manifested a double dependence on the instantaneous vapor pressure and on the rate of vapor pressure changes. To estimate this double dependence, the impulse frequency of a moist and dry cell during three consecutive “oscillation” periods was plotted in Fig. 5 as a function of both parameters of the humidity stimulus. Multiple regressions (*F* = *y_0_*+ *a (ΔPw/Δt)*+*b Pw*; where *F* is impulse frequency and *y_0_* the intercept of the regression plane with the *F* axis reflecting the height of the regression plane) were then calculated to determine the increase of response for the instantaneous vapor pressure (*b* slope) and its rate of change (*a* slope). The sign of regression slopes is positive for the moist cell and negative for the dry cell, i.e., an increase in both instantaneous vapor pressure and the rate of change led to a frequency increase in the moist cell but to a frequency decrease in the dry cell. The slopes are very similar with the other five pairs of a moist and dry cells tested in this manner. At 20°C, the mean gain value for the instantaneous vapor pressure (*b* slope) is +1.42 (impulses/s)/mbar for the 6 moist cells and –0.20 (impulses/s)/mbar for the 6 dry cells; the mean gain value for the rate with which the vapor pressure changes (*a* slope) is +2.50 (impulses/s)/(mbar/s) for the 6 moist cells and –0.70 (impulses/s)/(mbar/s) for the 6 dry cells. Thus in the moist cell, an increase of 1 impulse/s can be elicited either by increasing the instantaneous vapor pressure +0.7 mbar, provided the rate of change is constant, or by increasing the rate of vapor pressure change +0.4 mbar/s. In the dry cell, the corresponding values are –5.0 mbar and –1.4 mbar/s.

### Effect of Temperature on the Response to Oscillating Changes in Vapor Pressure

**Figure 6:**
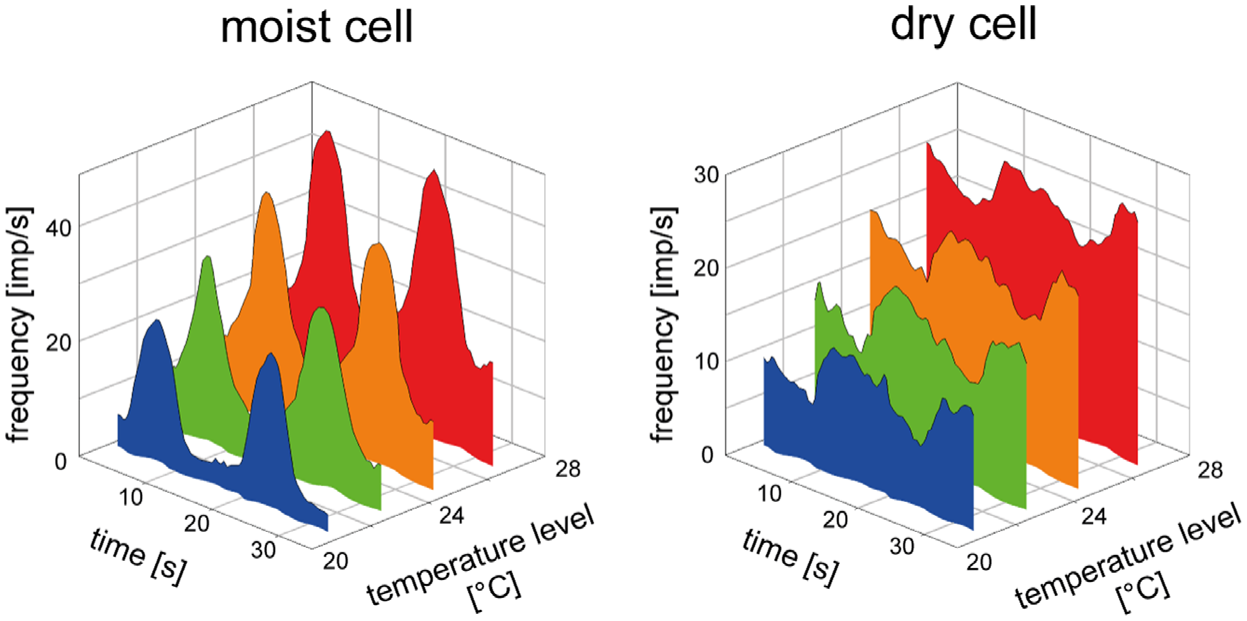
Humidity stimulation expressed as water vapour pressure. Plots of the time course of impulse frequency of a moist cell and a dry cell from the same sensillum during oscillating changes in vapor pressure at different temperature levels. With rising temperature, the oscillations in impulse frequency of the moist and dry cells shift upwards on the frequency scale.

Studying the effect of the temperature level on the discharge rates of the most and dry cells to slowly oscillating changes in vapor pressure requires to ensure that the temperature levels had actually been reached. After 5 min at a particular temperature level, there followed a series of a dozen oscillations in vapor pressure with constant amplitude and frequency. Then the temperature level was altered followed by the next series. This approach enabled several tests at different temperature levels on the 6 pairs of moist and dry cells. Fig. 6 shows a representative example. In both cells the discharge rates during vapor pressure oscillations rose with the temperature level at which the humidity oscillations took place. However, their oscillating impulse frequencies differed. In the moist cell the frequency oscillations were more rounded and smooth, and more rapid during the early phase of each rise or fall in humidity; moreover, the amplitudes were larger and the time course for a given temperature level encompassed larger segments of the frequency scale.

Despite the clear dependence of the cells’ discharge rates on the vapor pressure and air temperature, one cannot simply infer from this dependence that these two parameters represent the adequate stimulus. In itself, vapor pressure is a measure of the absolute humidity that is independent of temperature in this open-ended stimulus system. Nevertheless, the temperature dependence could disappear with the proper choice of the parameters involving humidity and temperature. Such a parameter is the relative humidity.

### Mechanical Hygrometers: Humidity Expressed as Relative Humidity

**Figure 7:**
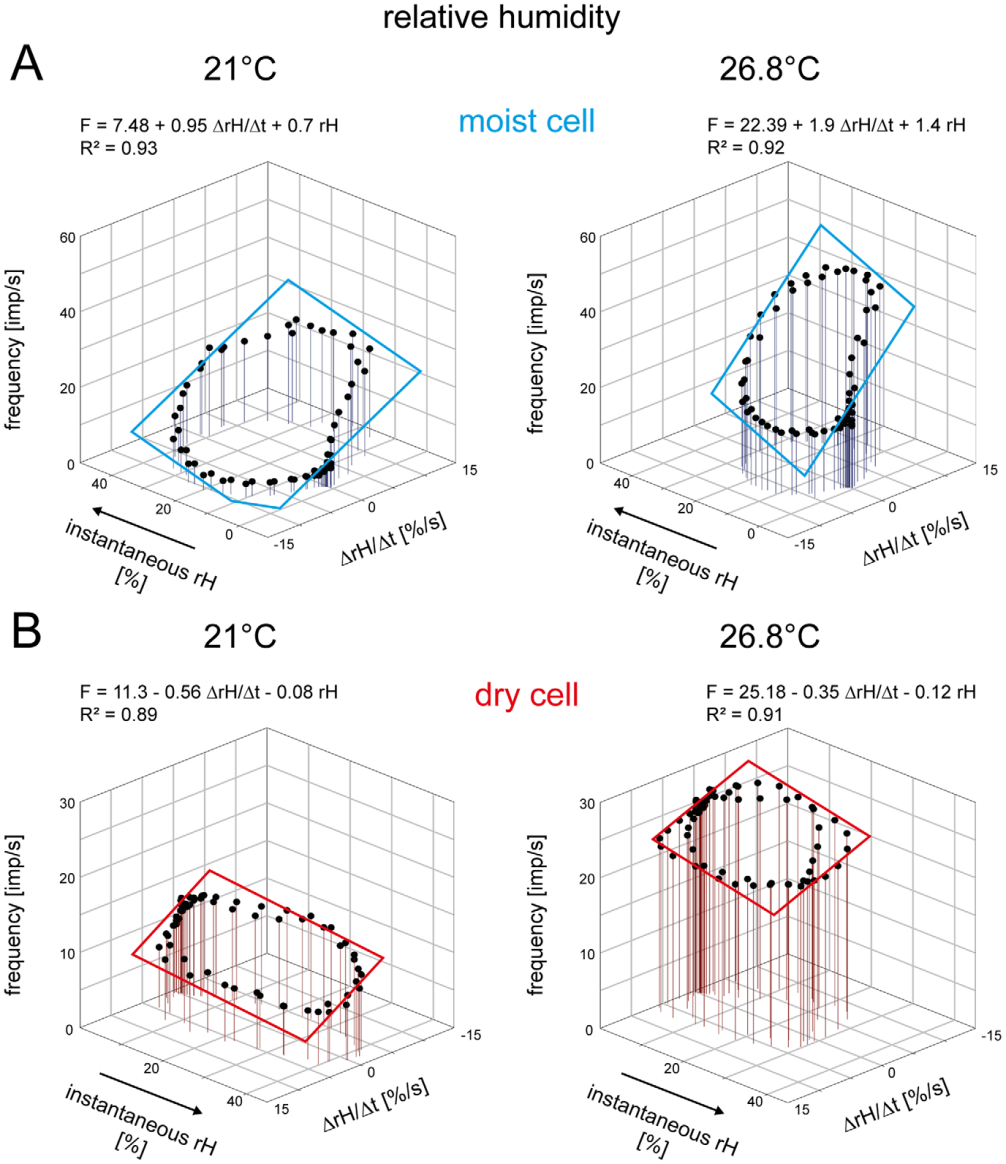
Humidity stimulation expressed as relative humidity. Impulse frequency of a moist cell (**A**) and a dry cell (**B**) from the same sensillum during oscillating changes in relative humidity at two different temperatures, plotted as function of instantaneous relative humidity and the rate with which the relative humidity changes. Regression planes [*F* = *yo*+*a* (Δ*rH*/Δt)+*b rH*; where *F* is the impulse frequency and *yo* is the intercept of the regression plane with the *F* axis reflecting is the height of the regression plane] were utilized to determine the gain values for instantaneous relative humidity (*b*-slope) and its rate of change (*a*-slope). Impulse frequency of the moist cell (**A**) increases linearly with rising instantaneous relative humidity and its rate of change, in the dry cell (***B***) with falling instantaneous relative humidity and its rate of change. *R^2^*, coefficient of determination; the number of points per plot was 60. *Arrows* point in the direction of increasing axis values. *F* impulse frequency, *rH* relative humidity.

In Fig. 7*A and B*, the responses of a pair of moist and dry cells to oscillating changes in vapor pressure at two different temperature levels (21°C and 26.8°C, respectively) were plotted as functions of the instantaneous relative humidity and its rate of change. The slopes of the regression planes indicate the simultaneous dependence on both parameters of the humidity stimulus. In general, the impulse frequency of the moist cell is higher at higher relative humidity and lower at the lower values (Fig. 7*A*). Conversely, the impulse frequency of the dry cell is higher at lower relative humidity and lower at the higher values (Fig. 7*B*). At a given relative humidity, impulse frequency of the moist cell is even higher when relative humidity is also rising (Fig. 7*A*), and in the dry cell when the relative humidity is also falling (Fig. 7*B*). Thus the effect of the instantaneous relative humidity on the hygroreceptor responses is reinforced by the rate of change of the relative humidity. These findings support the mechanical hygrometer model, which senses the relative humidity.

**Figure 8:**
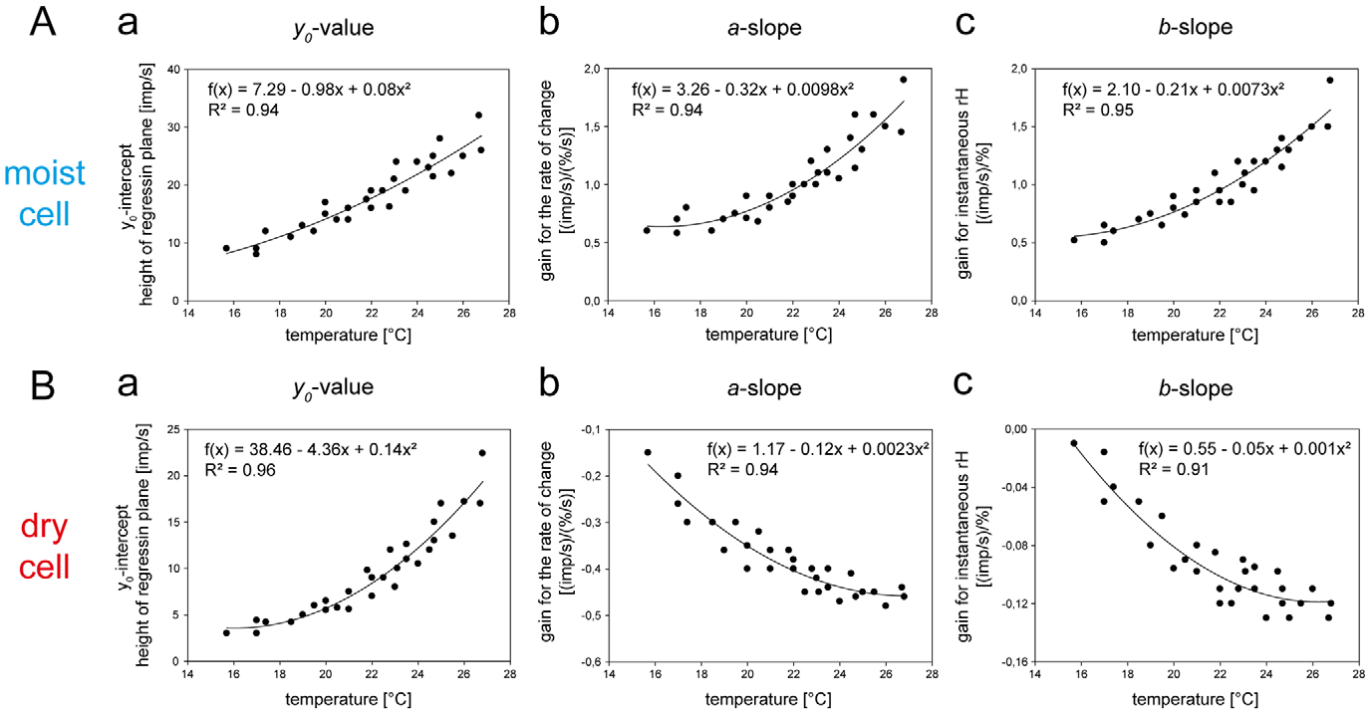
Humidity stimulation expressed as relative humidity. Effect of temperature on the 3 parameters of the regression plane utilized to determine the response characteristic of the moist cell and the dry cell to oscillating changes in relative humidity. **Aa** and **Ba**. *yo* intercept of the regression plane with the *F* axis reflecting the height of the regression plane plotted as function of temperature. **Ab** and **Bb**. Gain for the rate of change of the relative humidity plotted as function of temperature. **Ac** and **Bc**: Gain for the instantaneous relative humidity plotted as function of temperature. Relationships approximated by quadratic regressions [*f* = *yo*+*aT+aT^2^*]. *R^2^*, coefficient of determination; the number of points per plot was 30. *Arrows* point in the direction of increasing axis values. *F* impulse frequency, *rH* relative humidity.

For all 6 moist cells and 6 dry cells, the values of the three parameters of the regression planes (*y_0_* the height of the regression plane, *a* slope and *b* slope) were pooled and plotted in Fig. 8 against the temperature level. Quadratic functions were used to describe the dependence of each regression parameter on the temperature. The measurements show that in both cell types not only the response magnitude (as indicated by the height of the regression plane) increases with rising temperature (Fig. 8*A* *a,Ba*) but also the gain values for both components of the humidity stimulus (Fig. 8*A* *b,c*, *Bb,c*; the negative gain values reflect the downward direction of humidity change, yielding a rise in impulse frequency and specifying the dry cell).

The slope of a quadratic function varies continuously along the curve. Thus, no single slope value could be assigned to the entire segment of the quadratic function approximating the relationship between the regression parameters and temperature. Rather, slope values were provided by the first derivative of a given quadratic function at a regression parameter actually obtained from the regression plane. Each deviation therefore had its own corresponding slope. The mean slope for all deviations from a quadratic function was then computed. In the moist cell, this procedure gives an increase in the mean height of the regression plane by 1.9 impulses/s for each 1°C increase in temperature, and in the dry cell by 1.6 impulses/s. Thus in the moist cell an increase of 1 impulse/s can be elicited by raising the temperature for oscillations in the relative humidity by 0.5°C, in the dry cell by 0.6°C. The temperature dependence of the gain values was determined by the same method. In the moist cell, the mean gain for the instantaneous relative humidity increases by 0.10 (impulses/s)/%rH when the temperature rises 1°C, and in the dry cell by 0.04 (impulses/s)/%rH. The mean gain for the rate of humidity change increases in the moist cell by 1.10 (impulses/s)/(% rH/s) for a 1°C rise, and in the dry cell 0.02 (impulses/s)/(% rH/s). In the moist cell, it therefore takes a 10°C increase to increase the mean gain for the instantaneous relative humidity by 1 impulse/s; in the dry cell this increase is 25°C. To yield an average increase in a moist cell’s gain for the rate of change in the relative humidity of 1 impulse/s, the temperature must increase by 0.9°C, and in the dry cell by 50°C.

While in the moist cell the increase in the response magnitude to oscillations in the relative humidity with rising temperature does not agree with a mechanical hygrometer (Fig. 2*B* *a*, axis on right side), it does so in the dry cell (Fig. 2*B* *b*, axis on right side). Importantly, the inadequate response of the moist cell excludes relative humidity as the adequate stimulus.

### Evaporation Rate Detectors: Humidity Expressed as Saturation Deficit

**Figure 9:**
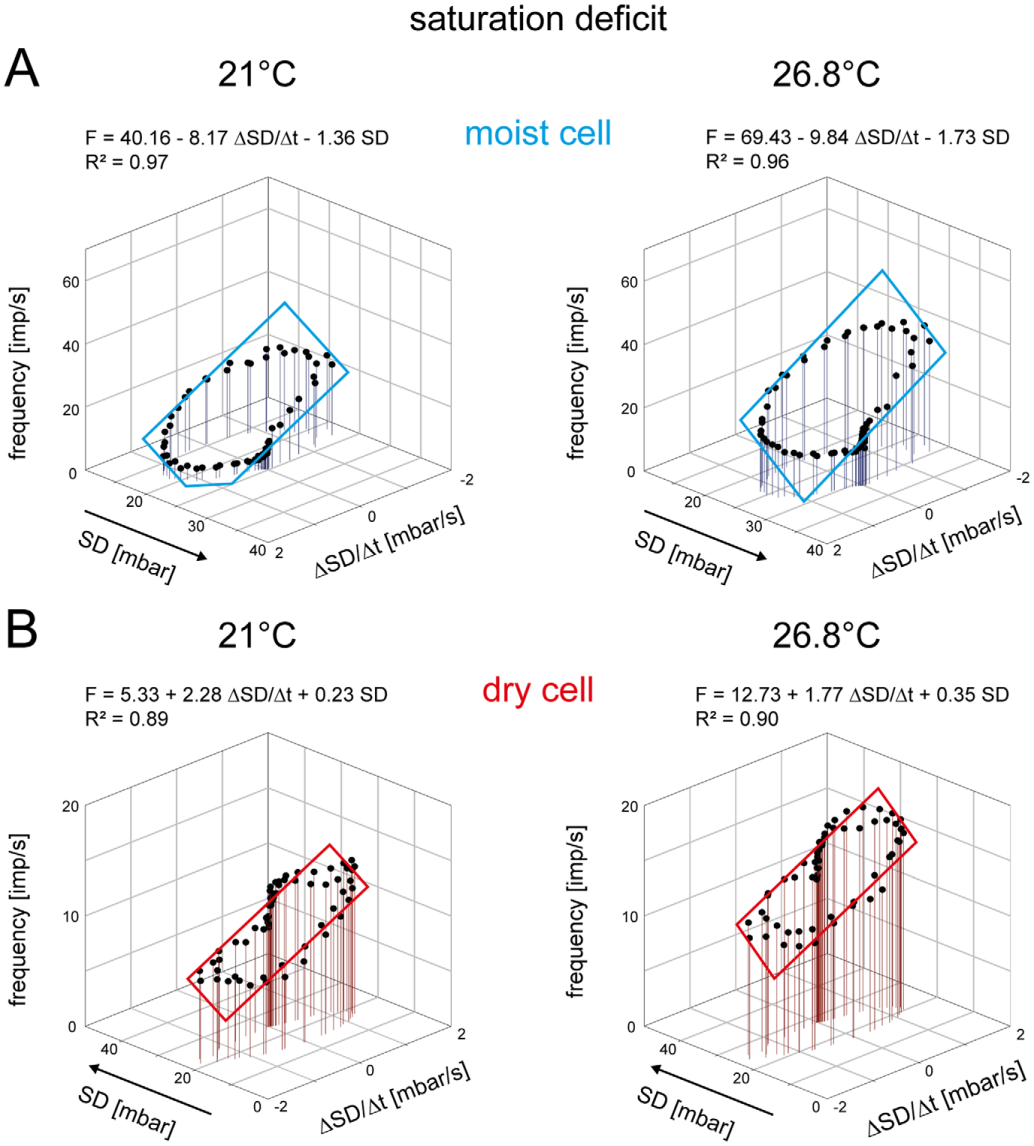
Humidity stimulation expressed as saturation deficit. Impulse frequency of the moist cell (**A**) and the dry cell (**B**) of Fig. 7 during oscillating changes in saturation deficit at two different temperatures, plotted as function of instantaneous saturation deficit and the rate with which the saturation deficit changes. Regression planes [*F* = *yo*+*a* (Δ*SD/*Δt)+*b SD*; where *F* is the impulse frequency and *yo* is the intercept of the regression plane with the *F* axis reflecting the height of the regression plane] were utilized to determine the gain values for the instantaneous saturation deficit (*b*-slope) and its rate of change (*a*-slope). Impulse frequency of the moist cell (**A**) increases linearly with rising instantaneous saturation deficit and its rate of change, in the dry cell (**B**) with falling instantaneous saturation deficit and its rate of change. *R^2^*, coefficient of determination; the number of points per plot was 60. *Arrows* point in the direction of increasing axis values. *F* impulse frequency, *SD* saturation deficit.

In Fig. 9*A and B*, the responses of the same pair of moist and dry cells shown in Fig. 7*A and B* for 21°C and 26.8°C were plotted as functions of the instantaneous saturation deficit and its rate of change. The slopes of the regression planes illustrate that the double dependence shown above for changes in the relative humidity still exists. The moist cell’s impulse frequency is higher at lower saturation deficit and lower at the higher values (Fig. 9*A*), while the dry cell’s impulse frequency is higher at the higher saturation deficit and lower at the lower values (Fig. 9*B*). At a given saturation deficit, impulse frequency of the moist cell is even higher when the saturation deficit is also falling (Fig. 9*A*), and in the dry cell, when the saturation deficit is also rising (Fig. 9*B*). Thus the responses of both hygroreceptor types to the instantaneous saturation deficit are reinforced by the rate with which the saturation deficit changes. These findings support the evaporation detector model, which senses the saturation deficit.

**Figure 10:**
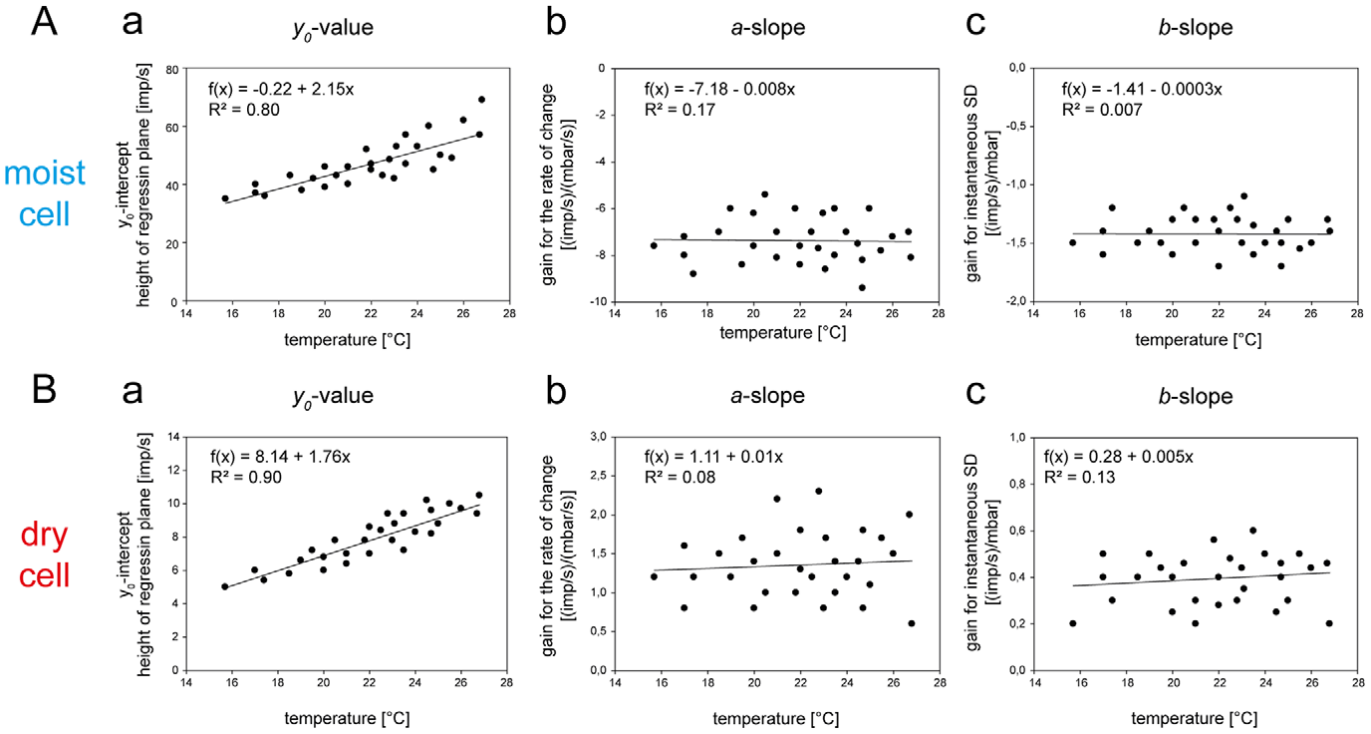
Humidity stimulation expressed as saturation deficit. Effect of temperature on the parameters of the regression plane utilized to determine the response characteristic of the moist cell (**A**) and the dry cell (**B**) to oscillating changes in the saturation deficit. *Aa* and *Ba*: *yo* intercept of the regression plane with the *F* axis reflecting the height of the regression plane plotted as function of temperature. **Ab** and **Bb**. Gain for the rate of change of the saturation deficit plotted as function of temperature. **Ac** and **Bc**. Gain for the instantaneous saturation deficit plotted as function of temperature. Relationships approximated by linear regressions [*f* = *yo*+*aT*]. *R^2^*, coefficient of determination; the number of points per plot was 30. *Arrows* point in the direction of increasing axis values. *F* impulse frequency, *SD* saturation deficit.

In Fig. 10*A and B*, for all 6 moist and dry cells, the values of the three parameters of the regression planes (*y_0_* height of the regression plane, *a*-slope and *b*-slope) were pooled and plotted against the temperature level. Linear regressions, which approximate the effect of temperature on the regression parameters, indicate that in both hygroreceptors the height of the regression plane increases with temperature (Fig. 10*A* *a,Ba*). In the moist cell, the regression slope shows an upward shift in the regression plane by 2.15 impulses/s for each degree °C the temperature is raised (Fig. 10*A* *a*), in the dry cell by 1.76 impulses/s (Fig. 10*B* *a*). Thus in the moist cell an increase in the response magnitude of 1 impulse/s can be elicited by raising the temperature during oscillating changes in the saturation deficit by 0.4°C, in the dry cell by 0.5°C. The mean gain values of both hygroreceptors for the instantaneous saturation deficit and the rate of change in saturation deficit display some variations (Fig. 10*A* *b,c* and *Bb,c*) but are not affected by temperature.

The lack of temperature dependence of the gain values is not at variance with hygroreceptors for the saturation deficit. The increase of the dry cell’s response magnitude with temperature (Fig. 10*B* *a*) supports the function of an evaporation rate detector (Fig. 2*C* *a*, right axis of the diagram). In the moist cell, in contrast, the increase in the response magnitude with temperature (Fig. 10*A* *a*) does not agree with a saturation-deficit receptor responding antagonistically to the dry cell (Fig. 2*C* *b*, right axis of the diagram).

### Psychrometer: Humidity Measured by Wet- and Dry-surface Thermoreceptors

**Figure 11:**
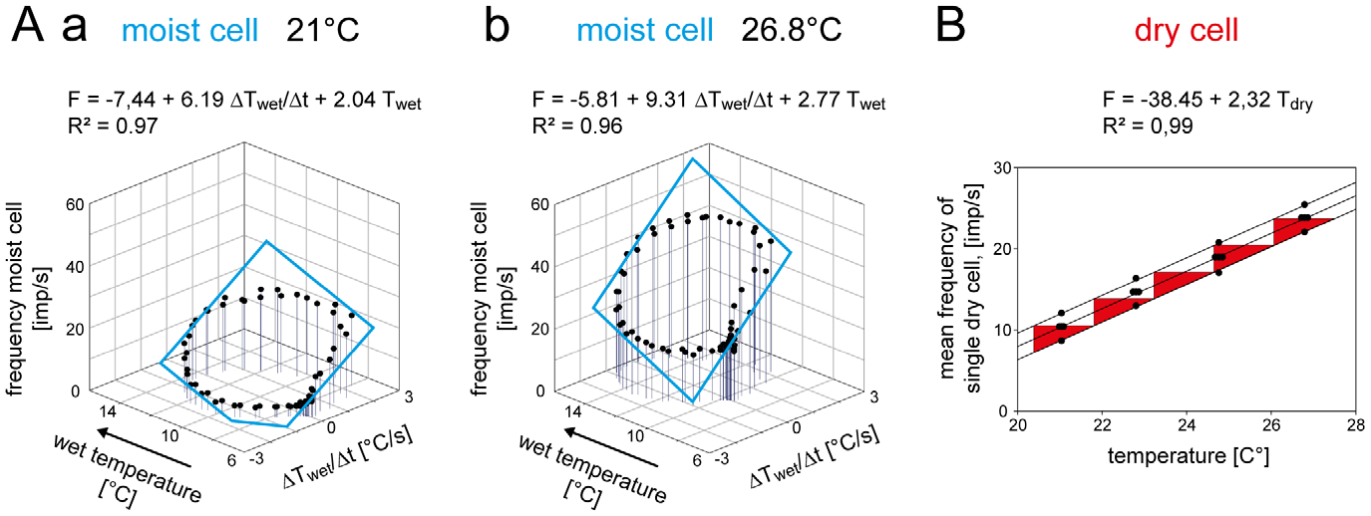
Humidity stimulation based on the wet- and dry-bulb principle. **A**. Impulse frequency of the moist cell (**A**) and the dry cell (**B**) of Fig. 7 during oscillating changes in the wet-bulb temperature at two different temperatures, plotted as function of instantaneous wet-bulb temperature and the rate with which the wet-bulb temperature changes. Regression planes [*F* = *yo*+*a* (Δ*Twet*/Δt)+*b Twet*; where *F* is the impulse frequency and *yo* is the intercept of the regression plane with the *F* axis reflecting the height of the regression plane] were utilized to determine the gain values for the instantaneous wet-bulb temperature (*b*-slope) and its rate of change (*a*-slope). Impulse frequency of the moist cell increases linearly with rising instantaneous wet-bulb temperature and its rate of change. **B**. Impulse frequency of the dry cell of Fig. 7*B* as function of the dry-bulb temperature (air temperature) during oscillating changes in the wet-bulb temperature at 4 different temperatures. Relationship approximated by linear regression [*f* = *yo*+*aT*]. Resolving power of impulse frequency for dry-bulb temperature (the number of discrete steps which impulse frequency can distinguish within the temperature range). The band width is determined by means and standard deviations of the responses to the dry-bulb temperature when testing the effect of oscillating changes in the wet-bulb temperature. The band enables 5 steps to be distinguished. *R^2^*, coefficient of determination; the number of points per plot in *A* was 30, in *B*, *5*. *Arrows* point in the direction of increasing axis values. *F* impulse frequency, *Twet* wet-bulb temperature, *Tdry* dry-bulb temperature.

Fig. 11*A* plots the responses of the moist cell shown in Figs. 7*A* and 9*A* for two temperature levels as a function of the instantaneous wet-bulb temperature and its rate of change. The slopes of the regression planes again show the established double dependence. The impulse frequency is higher at higher wet-bulb temperatures and lower at the lower values (Fig. 11*A* *a,b*), and at a given wet-bulb temperature the impulse frequency is even higher when the wet-bulb temperature is also rising (Fig. 11*A*). Thus, the moist cell’s responses to the instantaneous wet-bulb temperature are reinforced by the rate with which the wet-bulb temperature increases. These findings support evaporation cooling as the adequate stimulus.

**Figure 12:**
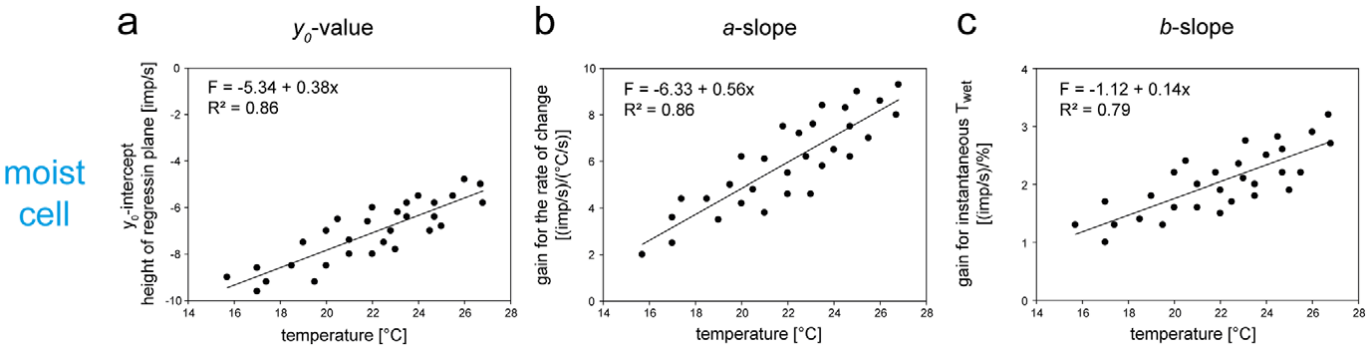
Humidity stimulation based on the wet-bulb principle. Effect of temperature on the parameters of the regression plane utilized to determine the response characteristic of the moist cell to oscillating changes in the wet-bulb temperature. **a**. *yo* intercept of the regression plane with the *F* axis reflecting the height of the regression plane plotted as function of the temperature level. **b**. Gain for the rate of change of the wet-bulb temperature plotted as function of temperature. **c**. Gain for the instantaneous wet-bulb temperature plotted as function of temperature. Relationships approximated by linear regressions [*f* = *yo*+*aT*]. *R^2^*, coefficient of determination; the number of points per plot was 30.

Fig. 12 pools and plots the values of the three parameters of the regression planes (*y_0_* height of the regression plane, *a*-slope and *b*-slope) against temperature for all 6 moist cells. Linear regressions were used to describe the relationships. As indicated by the regression slope, the height of the regression plane increases by 0.38 impulses/s for each 1°C rise (Fig. 12*a*). Thus an increase of 1 impulse per second can be elicited by raising the temperature for the wet-bulb temperature stimulation by 2.6°C. The mean gain for the instantaneous wet-bulb temperature increases by +0.56 (impulses/s)/°C for a rise of 1°C, the mean gain for the rate of wet-bulb temperature change increases by +0.14 (impulses/s)/(°C/s) (Fig. 12*b,c*). Accordingly, a 0.4°C increase is required to increase the mean gain for the instantaneous wet-bulb temperature by 1 impulse/s, the corresponding value to increase the mean gain for the rate of change in the wet-bulb temperature being 7.1°C.

Determining the amount of evaporation cooling from the value of the wet-bulb temperature requires a separate dry-bulb temperature measurement of the air temperature. Fig. 11*B* plots the dry cell’s responses shown in Figs. 7*B* and 9*B* as a function of the dry-bulb temperature. The function is linear and the coefficient of determination indicates that an average of 99% of the variation in impulse frequency can be explained by changes in the dry-bulb temperature. The values of frequency vary at the different temperatures from 6 to 8 impulses/s. This variation can be partially explained by temperature fluctuations during the oscillations in vapor pressure. The remaining variation reflects properties of the dry cell. The slope of the regression line indicates an average increase of 2.3 impulses/s when temperature rises by 1°C.

Based on the slope of the regression line and the scatter of the responses, the resolving power was determined by the maximum number of discrete steps that the dry cell’s impulse frequency can distinguish within the temperature range. To estimate the step numbers, other curves (both above and below the frequency vs temperature curve) were plotted. These curves enclose the deviation of the responses. Such a band reflects the degree of scatter. The number of stimulus steps drawn within the space enclosed by the deviations is 5. Step width is therefore 1.5°C. Resolving power was also calculated by the formula described in Material and Methods. The calculation yields a value of only 0.62°C. This is the difference which must separate two temperatures if the warmer is to be identified with 90% probability based on a single response of a single dry cell.

**Figure 13:**
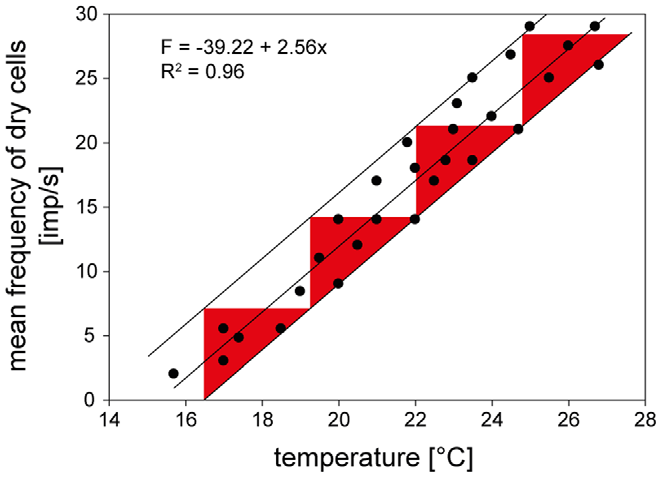
Humidity stimulation based on the dry-bulb principle. Impulse frequency of the dry cell of Fig. 7*B* as function of the dry-bulb temperature (atmospheric temperature) during oscillating changes in the wet-bulb temperature at different temperatures levels. Relationship approximated by linear regression [*f* = *yo*+*aT*]. Resolving power of impulse frequency for dry-bulb temperature (the number of discrete steps which impulse frequency can distinguish within the temperature range). The band encloses all responses throughout the range and enables 4 steps to be distinguished. *R^2^*, coefficient of determination; the number of points was 30. *F* impulse frequency.

The data from the single cell shown as an example in Fig. 11*B* are representative of all 6 dry cells. Impulse frequency is directly proportional to temperature. The pooled data in Fig. 13 provide a group estimate of the dry cell’s gain for the temperature. A linear regression indicates a mean rise in impulse frequency by 2.56 impulses/s for each degree °C the temperature is raised. The drawback of pooling, however, is also illustrated in Fig. 13: namely the wide scatter of the 30 data-points about the regression line used to approximate their course. Nevertheless, the coefficient of determination indicates that an average of 96% of the variation in impulse frequency can be explained by variation in the dry bulb-temperature. More important, the scatter is greater than that displayed by any single cell. This is because the scatter does not result from the deviations of the points from the regressions of individual dry cells. Rather, it reflects the variance in the slopes of these curves, i.e. the variance in gain for the temperature stimulus. Fig. 13 shows the band width that contains all responses of the 6 dry cells, illustrating the degree of scatter of individual frequency values about the mean response curve. The resolving power is determined by drawing the maximum number of steps through the space enclosed. This value is four, indicating a step width of about 3°C. When the method of calculating the resolving power was employed here, the value improved to 1.64°C. This is an estimate of the precision with which a dry cell with average gain can distinguish two temperatures with 90% probability.

These results verify the psychrometric transduction model. The moist cell acts as a wet-bulb thermometer, sensing the rate of water evaporation from the sensillum surface; and the dry cell acts as a dry-bulb thermometer sensing the ambient temperature.

## Discussion

### Temperature Dependence of the Humidity Response

Our primary finding is that the responses of the cockroach`s moist and dry cells to slow and continuous changes in the vapor pressure depend on temperature. Either parameter can be changed independently from the other, but both are related to the “wetness” or “dryness” of the atmosphere. Sensing these conditions is crucial to terrestrial arthropods as this helps to estimate the danger of water loss when emerging from cover. Clearly, the importance of atmospheric moisture as a factor affecting the water content of insects does not lie in the vapor pressure but in the relation between the amount present and the amount that could exist at saturation under the same conditions. This relation is expressed by the relative humidity or the saturation deficit. Nonetheless, relative humidity alone does not indicate the moisture conditions. An atmosphere with a relative humidity of 50% may indicate “dryness” when the temperature is high or “wetness” when the temperature is low (Fig. 1*D*). Since the vapor pressure is a measure of the quantity of water vapor present, the vapor pressure deficit satisfactorily expresses the gradient between the amount of moisture in the atmosphere and the saturation point, and indicates the evaporation rate at any temperature. To keep the evaporation rate constant, an insect must search for constant vapor pressure deficits rather than constant relative humidities. In other words, two different regions having the same relative humidity do not imply similar atmospheric water conditions unless temperatures are also identical. Regions with the same vapor pressure deficits, however, do influence evaporation rates in the same way whether temperatures are identical or not. The clear advantage of using saturation deficits rather than relative humidities in measuring the effects of atmospheric moisture is that saturation deficits combine the effects of humidity and temperature on water loss in one measure.

The alternative way to determine the evaporation rate is by the wet-bulb temperature depression. As vapor pressure decreases and temperature rises, the greater becomes the temperature depression and the power of air to desiccate. To determine the depression, two temperature measurements are needed: one by a temperature-sensitive cell with a “dry surface” and unaffected by cooling and one by temperature-sensitive cell with a “wet surface” cooled by evaporation. Depending on the sensitivity of the two thermoreceptive cells, a psychrometric humidity measurement may resolve smaller differences and detect higher rates of change than a mechanical hygrometer.

### Rapid vs Slow Humidity Changes

The traditional stimulation technique consisted of step-like up and down changes in the relative humidity. These changes were produced by shifting from a conditioning air stream at constant relative humidity to another at different constant relative humidity for several seconds and then back to the initial relative humidity for a recovery period of several minutes before the next step change. Due to the relatively high flow velocity of the two air streams, the transition for a step change was hardly longer than the time required for the second air stream to replace the first. As reported for the stick insect, transition time was about 20 ms [18], [19]. Unfortunately, in cockroach studies this period was not determined [7], [8]. Nevertheless, 100 ms seems to be a conservative approximation. During such a 100-ms step, a 30% change in the relative humidity would result in an average rate of change of 300%/s. Even though hygroreceptors can respond to such rapid humidity changes, with rates exceeding 100%/s [7], [8], [18], [19], it is less clear whether the moisture content of the sensillum would reach equilibrium so fast. Note that hair hygrometers have relatively long time constants. For temperatures between 0° and 30°C and relative humidities between 20% and 80%, a hair hygrometer indicates 90% of a rapid change within about 3 minutes. During humidity transients, therefore, the moisture of the sensillum will lag behind the humidity of the air wave front coming in contact with the sensillum. Thus, instantaneous values of the sensillum’s moisture will not correspond to instantaneous humidity values of the stimulating air stream.

As a result of the inability to determine instantaneous humidity values of the sensillum during transient changes, the difference in humidity between the two air streams used for conditioning and stimulation was taken as a parameter [7], [8], [18], [19]. Their choice, however, raises the question of their adequacy. If the rate of humidity change at the sensillum is so great upon switching the air streams that impulse frequency is no longer influenced by decreasing the duration of switching, but only by increasing the extent of the humidity change, then the humidity difference is adequate for describing the humidity stimulus. If decreasing the duration of switching does have an effect on impulse frequency, the values of impulse frequency assigned to any humidity change can only be too low. This is because shortening the switching period would raise impulse frequency. The hygroreceptive cells would be more sensitive than indicated, not less.

That there are values of the rate of humidity change where the humidity difference is definitely inadequate as a stimulus parameter can be shown simply by changing ambient humidity slowly and continuously. Studies on the stick insect and the cockroach [10], [20] have demonstrated that the impulse frequency of the moist and dry cells is not only a function of the instantaneous humidity but of its rate of change as well. Moreover, using slow and continuous humidity changes provides the possibility of assigning instantaneous humidity values of the air stream to the stimulus input of the hygroreceptors. In the present study, a 50% change was applied during a period of 5 s (Fig. 4*B*) with an average rate of change of 10%/s. Under these conditions the difference in the moisture of the sensillum and the humidity of the air stream will be insignificant. Thus, the humidity of the air stream represents the moisture of the sensillum or even the receptive sites of the moist and dry cells. Impulse frequency can then be set in a reasonably clear relationship to both instantaneous humidity and its rate of change.

### Metabolic Warming

When the sensillum was presented with slowly oscillating changes in vapor pressure, the oscillating responses of the moist and dry cells displayed clear differences in their dependence on temperature. Therefore, the possibility that the flow of heat may explain the differential temperature dependence of both cells is unlikely. The flow would have to be maintained while the air temperature is held constant. Since the necessary condition for thermal equilibrium is the equality of temperature, heat flow could persist at constant air temperature only when the sensillum temperature is different from the air temperature. One might envision a metabolic heater within the sensillum to hold the temperature difference. To account for the temperature dependence of the oscillating responses, heat flow would also have to vary with the temperature level utilized for humidity stimulation. Such a consideration seems quite unlikely. Furthermore, the speed of the air stream (2.5 m/s) crossing the antenna is very high relative to the diameter of the sensillum or even to that of the antenna – it is about 2*10^6^ sensillum diameters or 10^4^ antenna diameters per second. Relative to the sensillum, the amount of air contacting it per second is presumably also very great. This makes it very difficult to imagine how temperature differences could be maintained by the sensillum or even modulated by constant values of air temperature, and in doing so produce differences in heat flow sufficient to differently govern the impulse frequency of the moist and dry cells during oscillating humidity changes. Thus metabolic warming does not provide an adequate explanation for the temperature dependence described.

### Mechanical Hygrometer

The mechanical sensitivity of the cockroach’s moist and dry cells was originally demonstrated by slight lateral movements of the recording electrode [8]. Although it is straightforward to apply mechanical stimuli by nudging the recording electrode, the disadvantages are injury of the sensillum and lack of both reproducibility and quantification. Note that the moist and dry cells responded antagonistically to this kind of mechanical stimulation. While the activity of the former was increased by pulling the electrode and decreased by pushing, that of the latter was increased by push and decreased by pull. Subsequently, the effect of electrode movements was tested on hygroreceptive sensilla in the cricket [11]. Contrary to the cockroach, the moist cell’s activity is increased by electrode push and that of the dry cell by electrode pull. No attempt was made to explain this difference. One may suggest differences in the distribution of forces in the sensillum or in the pathway for force transmission, which may depend upon the localization and the strength of the force applied.

Basic to the investigation of the mechanical sensitivity was the attempt to determine the sensillum structures involved in the functioning of a mechanical hygrometer. The intimate association of the dendritic membranes with the cuticular wall appeared to be important. It was suggested that slight hygroscopic changes in the geometry of the wall may lead to deformations of the dendritic membranes and to voltage changes across them [8]. However, if the sensillum wall is so hygroscopic that it is able to withdraw water vapor from the air in quantities large enough to produce a graded mechanical effect on the dendrites, then it is unclear why it should not withdraw water from the inside as well. What would seal the cuticular wall off from the lymph cavity?

So far, there is no direct evidence that a hygroscopic mechanism is responsible for hygroreception. In the stick insect, however, the cold cell, located together with the moist cell and the dry cell in the same hygroreceptive sensillum, did produce a brief frequency increase when the vapor pressure was suddenly lowered without changing ambient temperature [21]. No temperature change would occur in the sensillum if its surface were dry. The most plausible explanation for the cold-cell’s response to a humidity drop is that evaporation of hygroscopically bound water from the sensillum surface lowers enthalpy. Another quantitative approach to demonstrate hygroscopic swelling and shrinking of the sensillum wall utilized atomic force microscopy. Nonetheless, high-resolution scans of the fine surface structures of the apical region of hygroreceptive sensilla in the honey bee revealed no change in the dimensions of the sensillum wall when ambient humidity was set at different levels [15]. This negative result is in line with the mechanical stimulation model in the form of changing air pressure [10]. The advantage of air pressure changes is avoiding direct contact with the sensillum surface so that there is no local compaction or physical impediment. The moist cell’s responses to both increasing humidity and increasing air pressure may reflect that swelling of the hygroscopic cuticle compresses the dendrites. Equally, the dry cell’s responses to decreasing humidity and decreasing air pressure may reflect that shrinking of the hygroscopic cuticle expands the dendrites. However, the responses to changes in air pressure are much weaker than those obtained during corresponding changes in vapor pressure. Thus, the mechanical parameters associated with air-pressure changes could only partially account for the responses of both types of hygroreceptors to humidity stimulation. The present study provides further arguments against the mechanical hygrometer model. While the moisture content and the geometry of hygroscopic structures such as human hair or cotton fibers are determined solely by the relative humidity and not affected by the temperature [22], [23], the responses of the cockroach’s moist and dry cells to the relative humidity are influenced by temperature.

**Figure 14:**
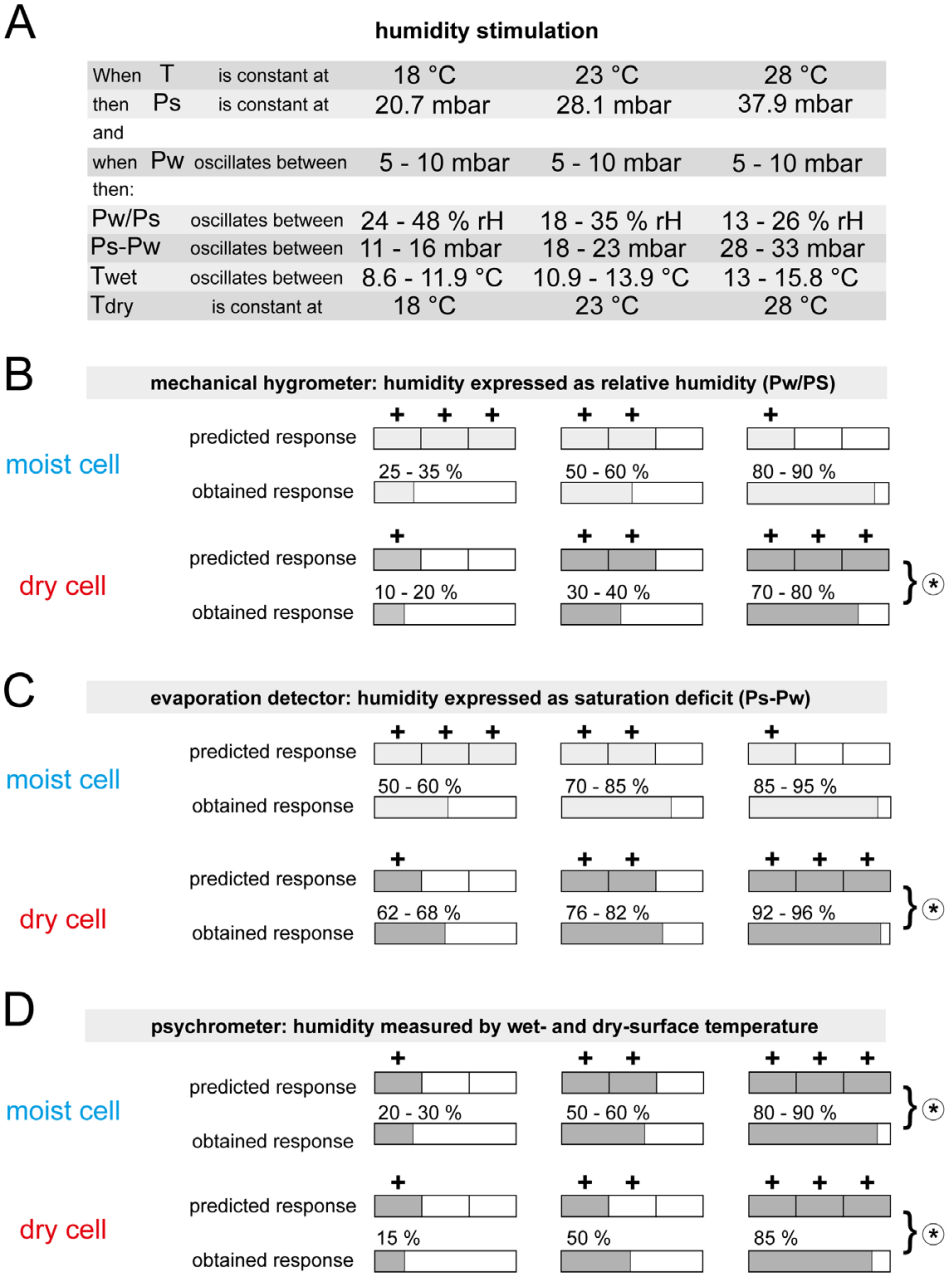
Summary of the electrophysiological analysis of the adequacy of the three humidity transduction models. Analysis is based on the specific predictions drawn from each of the models by determining the effect of three temperature levels on the responses of the moist and dry cells to oscillating changes in vapor pressure expressed as oscillation in relative humidity, saturation deficit or wet-bulb temperature. +,++and+++stand for categories of increasing response magnitude predicted by the models. Obtained response ranges of each cell are normalized to its response calculated for 30°C. **A**. Humidity stimulation. **B**. In the mechanical hygrometer swelling and shrinking of a hygroscopic sensillum wall due to changes in the relative humidity governs the response of the moist and dry cells. **C**. In the evaporation detector, humidity affects the lymph concentration outside the dendrites of the moist and dry cells, involving the saturation deficit. **D**. In the psychrometer, activity of the moist cell is initiated by evaporative cooling and actiovity of the dry cell by the temperature. *Asterisks* indicate correspondence between predicted and obtained responses. *Pw* water vapor pressure, *Ps* saturation water vapor pressure, *Pw/Ps* relative humidity, *Ps-Pw* saturation deficit, *T* temperature, *dry T* dry-bulb temperature, *wet T* wet-bulb temperature.

Since the present results are at variance with those obtained in previous studies, it would be relevant to update the effect of the temperature level on the responses of the moist and dry cells to different ways of expressing oscillating changes in vapor pressure. In Fig. 14*A*, three constant temperatures are combined with a constant amplitude oscillation in vapor pressure to generate different ranges of humidity stimulation. The entries in the matrix indicate the response magnitudes predicted by a particular transduction model and those actually obtained. Fig. 14*B* represents the data for the mechanical hygrometer model that serves to link the responses of the two hygroreceptors to the relative humidity. In the moist cell, the different rank order of the predicted and the obtained responses argues against relative humidity as the adequate stimulus, in the dry cell the similar rank order would confirm it. Since the relative humidity can only account for the responses of one of the two hygroreceptors, the mechanical hygrometer model is not applicable to humidity transduction.

### Evaporation Rate Detector

Evaporation is a form of water loss that is directly proportional to the saturation deficit of the surrounding air. A hygroscopic material is unnecessary. Rather, water from inside the sensillum is viewed as moving in one direction only, slowly towards the outside, where the water content is exposed to controlled evaporation in ambient air. The evaporation rate may change the lymph concentration of the surrounding dendrites of the hygroreceptive cells. To produce a humidity-dependent ion concentration in the lymph in a physiologically acceptable range at the dendritic level, however, this model requires an additional mechanism to hold the flow rate within narrow limits. The flow would have to be small enough to allow humidity to influence the concentration, but at the same time great enough to keep the concentration from being unphysiologically high under conditions normally encountered by the cockroach. The necessary resistance to the lymph flow would be furnished by the thin layer of lymph surrounding the dendrites and separating them from the cuticular wall, and by the poreless cuticular wall reducing the flow rate. An interesting observation that supports evaporation as a constitutive feature in hygroreception has been described for hygroreceptive sensilla of the bombycid moth [9]. When exposed to dry air, outward lymph flow results in reversible shortening (about 15% of the total length) of the dendritic processes. A further similarity is that one of the avenues proposed for lymph to reach the surface is the canal surrounding the dendrites.

The present study highlights the applicability of the evaporation model to the dry cell’s humidity responses. The example in Fig. 14*A* gives the range of the oscillating saturation deficit for the three different temperature levels. The responses predicted for the moist and the dry cells and actually obtained are shown in Fig. 14*C*. A comparison of the rank orders indicates that the dry cell but not the moist cell responds to the saturation deficit due to the rate of water evaporation from the sensillum surface.

### Psychrometer

In the psychrometer the cooling effect of water evaporation is used to measure humidity. As with the evaporation rate detector, no hygroscopic material is necessary. Water from inside will reach the surface, where it evaporates and cools the surface of the sensillum. The moist and dry cells are mainly thermoreceptors; the moist cell is associated with a “wet surface” and the dry cell with a “dry surface”. Dry air allows the water to evaporate, lowering the temperature of the wet-surface thermoreceptor below that of the surrounding air. The dry-surface thermoreceptor does not respond to evaporative cooling but indicates the temperature of the surrounding air. During slow changes in atmospheric humidity, the responses of the moist cell fit well with a wet-bulb thermometer and the dry cell with a dry-bulb thermometer.

The data described herein verifies the adequacy of the psychrometer model. Fig. 14*A* gives the range of the oscillating wet-bulb temperature for the three different temperature levels. The responses predicted and obtained for the moist cell are shown in Fig. 14*D*. A comparison of the rank order indicates that the moist cell responds to the wet-bulb temperature due to the cooling effect of water evaporation from the sensillum surface. The correspondence of the dry cell’s predicted and obtained responses verifies the function as a dry-bulb temperature detector.

### Sensing Temperature Changes Linked to Evaporation

The mechanisms involved in the psychrometer function however still remain elusive. Evaporation can be thought of as a slight discharge of aqueous solutions from the sensillum surface, which would in turn lead to the activation of temperature-sensitive transduction pathways. The sensitivity to mechanical manipulation is unclear. Evaporation may create mechanical forces in the form of fluid shear stress, pressure across or tension within the dendritic membranes that lead to membrane deformation as it has been suggested for the mechanical hygrometer model. The process of converting temperature changes due to evaporation requires fine-tuning of the transduction pathway. Because the hygroreceptor discharge is modulated by cooling and warming due to a change in evaporation, a steady receptor current must flow when the evaporation rate is in equilibrium with the ambient humidity. This current will balance between maximum and minimum discharge rates, and the temperature-sensitive transduction mechanism must account for this symmetric change in receptor current as well as for its maintenance.

### Conclusions and Suggestions for Future Work

The standard notion about humidity transduction overlooks the complicating effects of the dynamics of the humidity stimulus. While in previous studies the change in atmospheric humidity was made as close to a square wave as possible [7], [8], [18], [19], we developed a stimulation technique for testing slow and continuous humidity changes. The advantages of low rates of change were that instantaneous humidity values could be measured by means of UV-absorption instead of being calculated from calibrates values and also the moisture content of the hygroreceptive sensillum was allowed to reach equilibrium with the stimulating air stream. The results summarized in Fig. 14 verify the psychrometer model in which temperature changes due to evaporation is the adequate stimulus. However, we did not test the effect of altering the flow rate of the stimulating air stream. As the flow rate drastically affects the power of evaporation, experiments with slow and continuous changes in the saturation deficit at different flow rates would improve our knowledge on humidity transduction. Furthermore, although it is well established that temperature affects the responses of insect’s moist and dry cell to changes in the atmospheric humidity, nothing is known about the amplitudes and rates of both temperature and humidity changes that a running or even flying insect may encounter in the natural environment. Such data would be highly desirable.

## References

[1] Altner H, Schaller-Selzer L, Stetter H, Wohlrab I (1983) Poreless sensilla with inflexible socket. Cell Tissue Res 234: 279–307. 6196120 10.1007/BF00213769

[2] Altner H, Loftus R (1985) Ultrastructure and function of insect thermo- and hygroreceptors. Annu Rev Entomol 30: 273–295.

[3] Steinbrecht RA (1999) Bimodal thermo- and hygrosensitive sensilla. In: Harrison FW, Locke M, editors. Microscopic Anatomy of Invertebrates, Wiley-Liss, New York: 405–422.

[4] Tichy H, Loftus R (1996) Hygroreceptors in insects and a spider: humidity transduction models. Naturwissenschaften 83: 255–263.

[5] Yokohari F (1999) Hygro- and thermoreceptors. In: Eguchi E, Tominaga Y, editors. Atlas of Arthropod Sensory Receptors. Springer, Berlin Heidelberg New York, 191–210.

[6] Tichy H, Gingl E (2001) Problems in hygro- and thermoreception. In: Barth FG, Schmid A, editors. Ecology of Sensing. Springer, Berlin Heidelberg New York: 271–287.

[7] Yokohari F, Tateda H (1976) Moist and dry hygroreceptors for relative humidity of the cockroach, *Periplaneta americana* L. J Comp Physiol A. 106: 137–152.

[8] Yokohari F (1978) Hygroreceptor mechanism in the antennae of the cockroach *Periplaneta* . J Comp Physiol A 124: 53–60.

[9] Steinbrecht RA, Müller B (1991) The thermo−/hygrosensitive sensilla of the silkmoth, *Bombyx mori*: morphological changes after dry- and moist-adaptation. Cell Tissue Res 266: 441–456.

[10] Tichy H, Kallina W (2010) Insect hygroreceptor responses to continuous changes in humidity and air pressure. J Neurophysiol 103: 3274–3286. 20375249 10.1152/jn.01043.2009 PMC3206210

[11] Itoh T, Yokohari F, Tominaga Y (1984) Two types of antennal hygro- and thermoreceptive sensilla of the cricket, *Gryllus bimaculatus* (De Geer). Zool Science 1: 533–543.

[12] Becker D (1978) Elektrophysiologische Untersuchungen zur Feuchterezeption durch styloconische Sensillen bei *Mamestra brassicae* L (*Lepidoptera, Noctuidae*). Regensburg, 160.

[13] Loftus R (1976) temperature-dependent dry receptor on the antenna of *Periplaneta* – Tonic response. J Comp Physiol 111: 153–170.

[14] Tichy H (1979) Hygro- and thermoreceptive triad in antennal sensillum in the stick insect, *Carausius morosus* . J Comp Physiol 132: 149–152.

[15] Resch R, Ehn R, Tichy H, Friedbacher G (1998) In-situ investigation of humidity-induced changes on human hair and antennae of the honey bee, *Apis mellifera* L., by scanning force microscopy. Appl Phys A 66: 607–611.

[16] Loftus R, Corbière-Tichané G (1987) Response of antennal cold receptors of the catopid beetle, *Speophyes lucidulus* Delar., in sensilla with a lamellated dendrite. I. Response to suffen temperature change. J Comp Physiol A 161: 399–405.

[17] Diem K, Lentner C (1968) Wissenschaftliche Tabellen. J.R. Geigy AG, Basel.

[18] Tichy H (1987) Hygroreceptor identification and response characteristics in the stick insect *Carausius morosus* . J Comp Physiol A 160: 43–53.

[19] Tichy H, Loftus R (1990) Response of moist-air receptor on antenna of the stick insect, *Carausius morosus*, to step changes in temperature. J Comp Physiol A 166: 507–516.

[20] Tichy H (2003) Low rates of change enhance effect of humidity on the activity of insect hygroreceptors. J Comp Physiol A 189: 175–197. 10.1007/s00359-003-0397-z 12664093

[21] Tichy H (2007) Humidity-dependent cold cells on the antenna of the stick insect. J Neurophysiology 97: 3851–3858. 10.1152/jn.00097.2007 17392413

[22] Crank J (1960) Diffusion of fiber-forming substances. In: Hearle, JWS, Peters, RH editors. Moisture in Textiles. Butterworths Publications Ltd., London, 106–122.

[23] Urquhart AR (1960) Sorption Isotherms. In: Hearle JWS and Peters RH, editors. Moisture in Textiles. Butterworths Publications Ltd., London, 14–32.

